# Heteroaryldihydropyrimidine (HAP) and Sulfamoylbenzamide (SBA) Inhibit Hepatitis B Virus Replication by Different Molecular Mechanisms

**DOI:** 10.1038/srep42374

**Published:** 2017-02-13

**Authors:** Zheng Zhou, Taishan Hu, Xue Zhou, Steffen Wildum, Fernando Garcia-Alcalde, Zhiheng Xu, Daitze Wu, Yi Mao, Xiaojun Tian, Yuan Zhou, Fang Shen, Zhisen Zhang, Guozhi Tang, Isabel Najera, Guang Yang, Hong C. Shen, John A. T. Young, Ning Qin

**Affiliations:** 1 Roche Pharma Research and Early Development, Chemical Biology, Roche Innovation Center Shanghai, Shanghai, 201203, China; 2 Roche Pharma Research and Early Development, Medicinal Chemistry, Roche Innovation Center Shanghai, Shanghai, 201203, China; 3 Roche Pharma Research and Early Development, Immunology, Inflammation and Infectious Diseases Discovery and Translational Area, Roche Innovation Center Shanghai, Shanghai, 201203, China; 4 Roche Pharma Research and Early Development, Immunology, Inflammation and Infectious Diseases Discovery and Translational Area, Roche Innovation Center Basel, CH-4070 Basel, Switzerland

## Abstract

Heteroaryldihydropyrimidine (HAP) and sulfamoylbenzamide (SBA) are promising non-nucleos(t)ide HBV replication inhibitors. HAPs are known to promote core protein mis-assembly, but the molecular mechanism of abnormal assembly is still elusive. Likewise, the assembly status of core protein induced by SBA remains unknown. Here we show that SBA, unlike HAP, does not promote core protein mis-assembly. Interestingly, two reference compounds **HAP_R01** and **SBA_R01** bind to the same pocket at the dimer-dimer interface in the crystal structures of core protein Y132A hexamer. The striking difference lies in a unique hydrophobic subpocket that is occupied by the thiazole group of **HAP_R01**, but is unperturbed by **SBA_R01**. Photoaffinity labeling confirms the **HAP_R01** binding pose at the dimer-dimer interface on capsid and suggests a new mechanism of HAP-induced mis-assembly. Based on the common features in crystal structures we predict that T33 mutations generate similar susceptibility changes to both compounds. In contrast, mutations at positions in close contact with HAP-specific groups (P25A, P25S, or V124F) only reduce susceptibility to **HAP_R01,** but not to **SBA_R01**. Thus, HAP and SBA are likely to have distinctive resistance profiles. Notably, P25S and V124F substitutions exist in low-abundance quasispecies in treatment-naïve patients, suggesting potential clinical relevance.

It is estimated that hepatitis B virus (HBV) causes 4 million acute infections and 686,000 deaths annually worldwide. More than 240 million individuals suffer from chronic HBV infection, which is a high risk factor for liver cirrhosis and hepatocellular carcinoma^1^. Although HBV vaccine has contributed successfully to the decline of prevalence of HBV infection, a significant number of populations in developing countries have limited access to it. In addition, some populations respond poorly to the vaccine^2^. In the past few decades, two formulations of interferon alpha and five nucleos(t)ide analogues were approved for HBV therapy. However, low rate of HBsAg loss, adverse effects particularly associated with interferon treatment and resistance emergence prevent the cure of HBV infection (ultimate elimination of cccDNA-mediated persistence)^3^. To meet the unmet medical needs, new generation anti-HBV agents are under development to inhibit viral targets other than the viral polymerase^4^.

The HBV core protein (also known as HBcAg or Cp) is an essential component and regulator of the HBV life cycle^5^,^6^. The full length core protein Cp183 or its N-terminal domain Cp149 predominantly assembles into a T = 4 icosahedral capsid^7^,^8^. Due to its critical roles in capsid assembly, pregenomic RNA packaging, cccDNA maintenance^9^,^10^ and suppression of innate immunity^11^,^12^, the HBV core protein has been recognized as an attractive antiviral target^13^,^14^,^15^,^16^,^17^. Different chemical classes of inhibitors targeting the HBV capsid are under development: heteroaryldihydropyrimidines (HAPs) and sulfamoylbenzamides (SBAs) (Fig. 1a). The first HAP compound **Bay 41-4109**^13^,^18^ promotes core protein assembly and leads to irregular particles^19^ and eventually causes core protein degradation^13^. Another HAP compound **HAP1**, causes large pleiomorphic morphology of aberrant capsids^20^,^21^. Interestingly, the low resolution crystal structure of **HAP1** treated capsids still shows the adoption of icosahedral symmetry with global changes^22^. The detailed interactions between assembled capsid and different classes of inhibitors remain elusive due to low resolution of the structures and/or low occupancy of the inhibitors. Recently published high-resolution complex structures^23^,^24^, using a non-assembled core protein mutant Y132A as a surrogate, greatly facilitate structure-based drug design of next-generation HAPs^24^. However, mechanism of abnormal core protein assembly triggered by HAPs is still not fully understood. In addition to HAP, SBA series was identified through a cell-based screening and disrupted the pgRNA encapsidation^25^. In comparison to HAPs, SBAs do not result in significant reduction of core protein. However, whether SBA directly targets core protein or the interface of capsid, polymerase and pgRNA is still unknown^25^.

To evaluate potential differences of these two classes of core protein modulators, we characterize the mechanisms of HAP and SBA and present the crystal structures of two reference compounds **HAP_R01** and **SBA_R01** in complex with the core protein mutant hexamer (Y132A). Based on structural and biophysical data, we predict amino acid substitutions that will alter viral susceptibility to HAP and SBA. Furthermore, we also identify clinical substitutions from treatment-naïve patients that are predicted to be non-susceptible to HAP, but still sensitive to SBA. Our results provide important new insights into how HAP and SBA interact with HBV capsid and may shed light on next generation of capsid inhibitors should HBV mutants emerge following treatment with classical HAPs.

## Results

### HAP and SBA inhibit viral DNA replication, but induce different effects on capsid assembly

**Figure 1:**
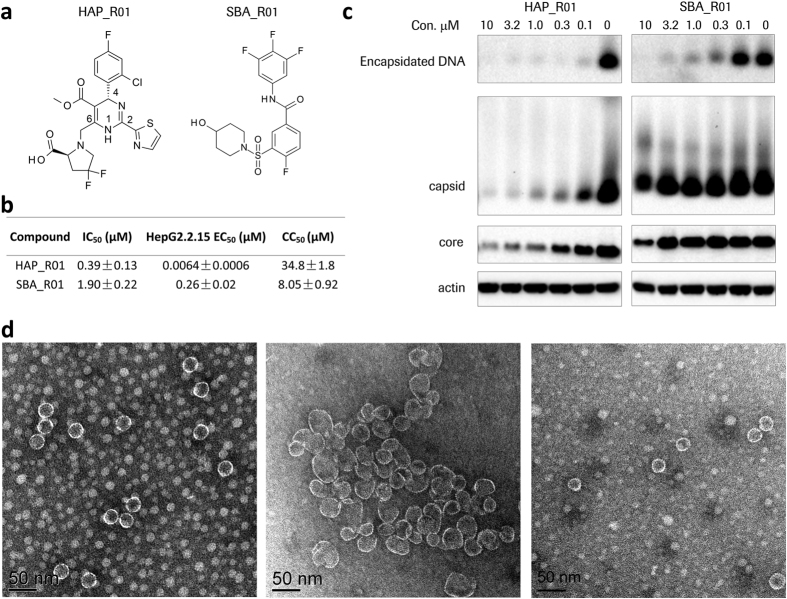
Differentiation of HAP_R01 and SBA_R01. (**a**) Chemical structures of HAP and SBA reference compounds. (**b**) Activity of HAP and SBA compounds. Shown are mean IC_50_ values of biochemical quenching assay, mean EC_50_ values of HepG2.2.15 antiviral assay and mean CC_50_ values of cytotoxicity experiment (±standard deviation). Each is from three independent experiments. (**c**) Electrophoresis of core particles in HepG2.2.15 cell lysate. Both tested compounds are effective in reducing encapsidated DNA. **HAP_R01**-treated cells show concentration-dependent reduction of capsid level on a native agarose gel and core protein level on a denatured gel. In contrast, **SBA_R01** does not diminish either capsid level or core protein level at non-cytotoxic concentrations. The cropped DNA and native gels are shown here for clarity. The full-length blots for the DNA and native gels are presented in Supplementary Fig. S13. Actin is the loading control. (**d**) Electron micrographs of compound-treated core protein assembly at the 1:1 dimer-to-compound ratio. Left: capsid control induced by 250 mM NaCl. Middle: 5 μM core protein dimer incubated with 5 μM **HAP_R01**. Right: 5 μM core protein dimer incubated with 5 μM **SBA_R01**. Black scale bar indicates 50 nm.

After rounds of structure activity relationship (SAR) exploration, we discovered a potent heteroaryldihydropyrimidine (HAP) inhibitor **HAP_R01.** In parallel, we identified **SBA_R01**, a novel capsid assembly modulator belonging to sulfamoylbenzamide (SBA) series through a focused library screening (Fig. 1a). Core protein assembly was monitored by the fluorescence quenching of BoDIPY- labelled Cp150. **HAP_R01** and **SBA_R01** can effectively reduce the fluorescence signal with a mean IC_50_ of 0.39 μM and 1.90 μM, respectively (Fig. 1b). The reduction of viral DNA level in the supernatant of HepG2.2.15 cell cultures was detected by quantitative PCR. Single digit nanomolar potency (EC_50_: 6.4 nM) is achieved for **HAP_R01**. As an early stage reference compound, **SBA_R01** shows a reasonable potency with EC_50_ of 260 nM. The distinct chemical structures and different activities of **HAP_R01** and **SBA_R01** intrigued us to further investigate the mechanism(s) of HAP and SBA in inhibiting HBV DNA replication. The levels of core protein, capsid and encapsidated viral DNA in HepG2.2.15 cell lysate were examined by electrophoresis after compound treatment. Both **HAP_R01** and **SBA_R01** can reduce the viral DNA level co-migrating with HBV capsid. **HAP_R01** is more effective in depleting capsid-associated DNA than **SBA_R01**, consistent with the higher potency **HAP_R01** in core protein assembly and viral DNA reduction (Fig. 1b,c). **HAP_R01** at 0.1 μM concentration induces significant degradation of capsid and total core protein, which is in agreement with of **Bay 41-4109**^13^. In contrast, **SBA_R01** at concentration up to 3.2 μM (12 times of EC_50_) does not cause the depletion of either core protein or capsid, similar to other SBAs^25^. The cytotoxicity CC_50_ value **SBA_R01** in HepG2.2.15 cells is about 8 μM (Fig. 1b), which could explain the decrease of core protein and capsid when treated with 10 μM of **SBA_R01** (Fig. 1c).

To evaluate the effect of **HAP_R01** or **SBA_R01** on the core protein assembly products, negative staining electron microscopy (EM) was performed. The NaCl-treated control appears as regular sphere with a diameter of about 30 nm. **HAP_R01** leads to large irregular particles in the 50 to 100 nm scale. At the dimer: **HAP_R01** ratio of 1:1, a small percentage of 30 nm particles are still visible (Fig. 1d). At the 1:4 stoichiometry, core proteins dominantly assemble into larger envelopes with open edges (Supplementary Fig. S1). In contrast, **SBA_R01** does not promote the enlarged assembly at both tested concentrations. The distinct morphologies of HAP and SBA-treated core protein assembly prompt us to investigate whether they targeted the same site of the core protein.

### Crystal structures of HBV core protein Y132A hexamer in complex with HAP_R01 and SBA_R01

**Figure 2:**
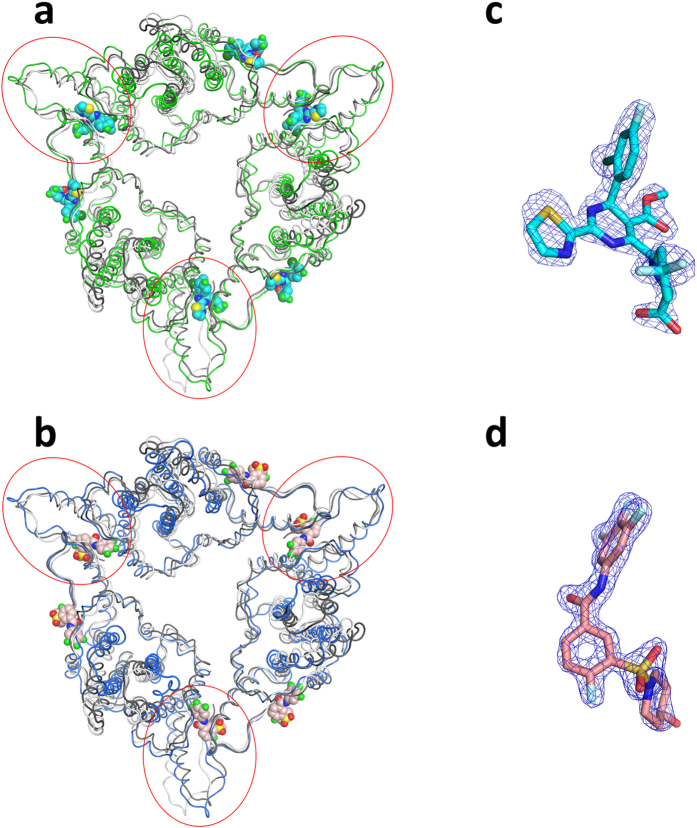
Crystal structures of Y132A hexamer in complex with reference compounds. (**a**) Y132A-**HAP_R01** structure represented in green ribbon is superimposed onto two types of hexamers from wild type (WT) capsid (1QGT). The hexamer around the quasi three-fold axis (chains ABCDBA) is shown in white ribbon. The hexamer around the three-fold axis (chains CDCDCD) is shown in grey ribbon. Six bound **HAP_R01** molecules are shown as space-filling models (cyan for carbon atoms). The red circles highlight three compounds at the dimer-dimer interfaces. (**b**) Y132A-**SBA_R01** structure represented in blue ribbon is superimposed onto two types of hexamers from 1QGT. White and grey ribbons are displayed as in (**a**). The carbon atoms of six bound **SBA_R01** molecules are colored in pink. (**c**) The simulated annealing omit Fo-Fc map (blue mesh) of **HAP_R01** (cyan stick) between the B-C interface is contoured at σ = 4 (**d**) The simulated annealing omit Fo-Fc map (blue mesh) of **SBA_R01** (pink stick) between the B-C interface is contoured at σ = 6.

To identify the specific interaction modes between core protein and **HAP_R01** or **SBA_R01**, we crystallized the Cp149 mutant Y132A, which remains as dimer in high salt solution and destabilizes Cp149 capsid^26^. Cp149_Y132A crystallizes in three forms: (A) open triangle, formed by trimer of dimers^27^, (B) closed triangle, mimicking the three CD dimers around the threefold axes of HBV capsid (Fig. 2)^28^, and (C) spike-contact packing (discovered in this work). The crystal in closed triangle form serves as a robust crystallization system for structure-based drug design. The crystal structures of core protein with **HAP_R01** or **SBA_R01** were determined at 1.95 Å or 1.69 Å resolutions, respectively (Supplementary Table S1). In both cases, six compounds bind to one hexamer. Each of the three dimer-dimer interfaces accommodates one compound. The other three compounds are located at similar pockets created by the dimers and their symmetry-related counterparts. When superimposing Y132A-**HAP_R01** hexamer onto the hexamer around the icosahedral three-fold axis (chains CDCDCD from 1QGT), the root-mean-square deviation (RMSD) for α carbon is 3.42 Å, which is slightly larger when superimposing to the hexamer (chain ABCDBA) around the quasi three-fold axis (3.67 Å) (Fig. 2a); the corresponding RMSD values for Y132A-**SBA_R01** have the same trend (3.74 Å and 3.94 Å, respectively) (Fig. 2b). Because Y132A is an assembly-deficient mutant^26^ representing a subset of WT dimer conformation^27^, yet **HAP_R01** and **SBA_01** have drastically different effects on WT dimer assembly (Fig. 1d and Supplementary Fig. S1), the co-crystal structures described here presumably resemble the intermediate states of different compound-induced core protein assembly. The interactions between the compounds and the Y132A mutant derived from the high resolution crystal structures have successfully guided lead optimization for HBV capsid inhibitors^24^ (see further evaluation in photoaffinity results and discussion). The simulated annealing omit Fo-Fc maps show that the absolute configuration of both compounds is well defined in the electron densities (Fig. 2c,d).

### Comparing the ligand binding sites of HAP_R01 and SBA_R01 on Y132A hexamer

**Figure 3:**
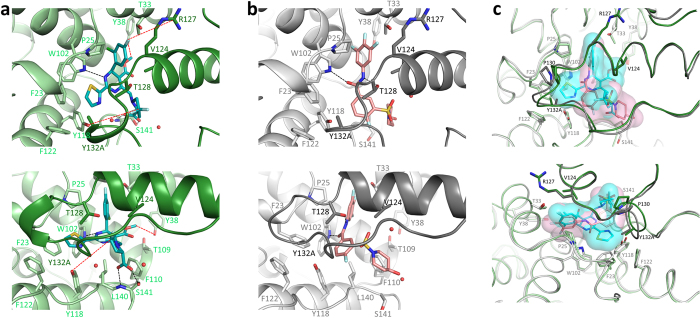
Comparing the binding modes of HAP_R01 and SBA_R01. The compound binding pocket at B-C interface is shown as an exemplary site. Chain B and Chain C of Y132A-**HAP_R01** structure are colored in light green and dark green respectively. Chain B and Chain C of Y132A-**SBA_R01** structure are colored in light grey and dark grey respectively. Key residues in the concave and the cap are shown in sticks. Black dash lines indicate hydrogen bonds between compounds and core protein. (**a**) Y132A-**HAP_R01** structure. Carbon atoms of **HAP_R01** are represented in cyan stick. Red dash lines mark the potential labeling reactions between photoprobes and their labeling residues in WT capsid. (**b**) Y132A-**SBA_R01** structure. Carbon atoms of **SBA_R01** are represented in pink stick. (**c**) Overlay of **HAP_R01** and **SBA_R01** binding pockets. **HAP_R01** and **SBA_R01** are also highlighted in transparent molecular surface with cyan and pink color respectively. Upper panel: top view from spike to contact domain. Lower panel: selected side view for clear representation of ligand-protein interactions.

**HAP_R01** and **SBA_R01** bind to the same dimer-dimer interface in the Y132A structures. These two compounds exhibit similar binding modes with small but notable differences. Taken the **HAP_R01** at the BC interface as an example, the binding pocket has a contact surface of 474.9 Å^2^, approximately 60% of which is contributed by the B chain. For **SBA_R01**, the contact surface is 402.3 Å^2^ with 60% of the area from the B chain as well. Since the B chain provides more contact surface area to ligands, we term the B chain part of the pocket as “concave” and the C chain part of the pocket as “cap”. In **HAP_R01** bound structure, the concave is created by F23, P25 from loop 2, D29, L30, T33, L37 from helix 2, W102, I105, S106, T109, F110 from helix 4, Y118, F122 from helix 5, and I139, L140, S141 from loop 6 of chain B. The cap is composed of V124, W125, R127 from helix5 and T128, P129, Y132A, R133, P134 from loop 6 of chain C (Fig. 3). The binding concave and cap of **SBA_R01** pocket are composed of similar set of residues, except that L37, T109, F122 from chain B and R133 from chain C do not directly contact **SBA_R01**. When superposing Cα of pocket residues with apo Y132A structure (4BMG^28^), **HAP_R01** pocket and **SBA_R01** pocket give RMSDs of 0.81 Å and 0.59 Å, respectively (Supplementary Fig. S2).

The halogenated phenyl group at the 4^th^ position of **HAP_R01** superimposes with that of the aniline of **SBA_R01**. Both insert into a deep halogen-binding subpocket formed by the same set of residues, i.e. P25, D29, L30, T33, W102, I 105, S106 from the concave, and V124, R127, T128 from the cap (Fig. 3c, Supplementary Fig. S3). Both compounds form a key hydrogen bond with W102, where the dihydropyrimidine 3-nitrogen of **HAP_R01** and the benzamide oxygen of **SBA_R01** act as the hydrogen bond acceptors. The 2^nd^-positioned thiazole group and the 5th-positioned methoxycarbonyl group of **HAP_R01** grow into two opposite directions and remain roughly co-planar with the dihydropyrimidine core. The carbonyl oxygen is solvent accessible, whereas the thiazole occupies a largely hydrophobic subpocket composed of F23, P25, W102, Y118, F122 from the concave, and T128, P129, Y132A from the cap. The aromatic residues Y132, F23, Y118 and F122 at the dimer-dimer interface are crucial for icosahedral capsid assembly^7^,^8^. Y132A^26^ or F23A^28^ mutation is known to disrupt capsid formation. Placing a bulky function group like thiazole, **HAP_R01** can induce local rearrangements at F23 and Y118 of the concave and the proline-rich loop 6 (P130, A131, Y132A) of the cap. Unlike the protruding thiazole of **HAP_R01**, **SBA_R01** does not occupy this subpocket. The gem-difluoro group on the pyrrolidine ring of **HAP_R01** makes contacts with W125 and P134 from the cap, while the carboxyl group forms strong bidentate interaction with the backbone nitrogen and the sidechain oxygen of S141 on the edge of the concave. In contrast, the piperidyl group of **SBA_R01** is fully accessible to solvent. The water-mediated network is well-defined in both structures. Noteworthily, the benzamide core of **SBA_R01** does not superimpose with the dihydropyrimidine core of **HAP_R01**, suggesting the dimer-dimer interface of HBV core protein can be targeted by different chemical scaffolds.

### Identification of the HAP binding site on capsid assembly by photoaffinity labeling

**Figure 4:**
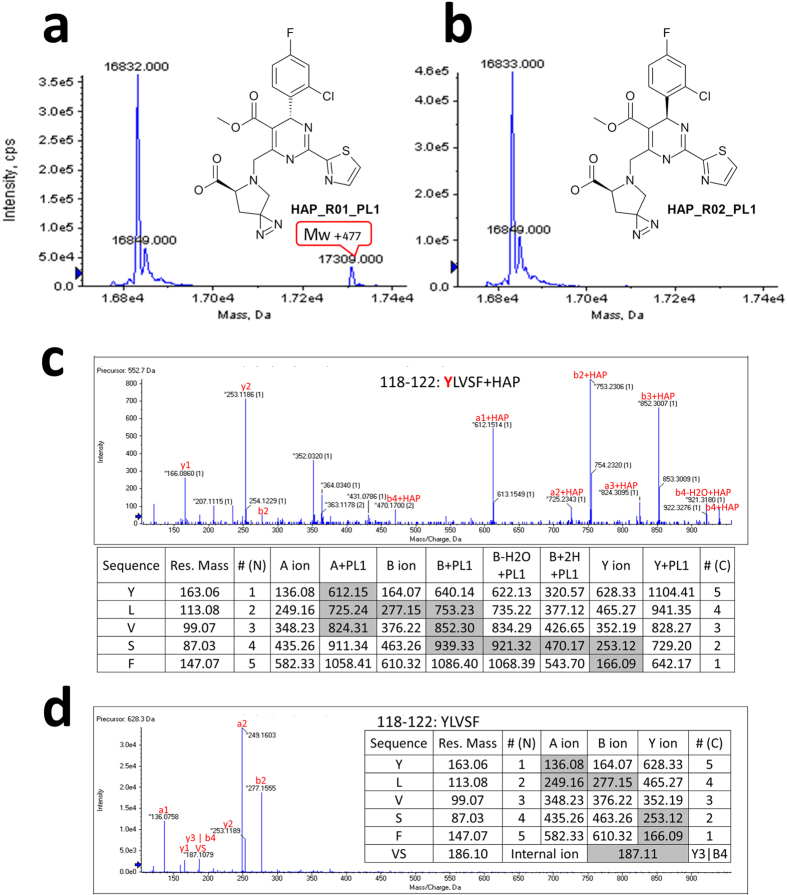
Identification of photoaffinity labeling site Y118 by HAP_R01_PL1. (**a**) Intact mass measurement of the active photolabel **HAP_R01_PL1**-treated sample. Mw increase of 477 is highlighted in red box indicating the covalent labeling of **HAP_R01_PL1** on core protein assembly. (**b**) Intact mass measurement of the control photolabel **HAP_R02_PL1**-treated sample. (**c,d**) MS2 data of pepsin-digested peptide 118–122: YLVSF from photoaffinity label-treated samples. Inlet tables show the calculated mass of fragmented ions. Grey shading denotes observed fragment ions. (**c**) **HAP_R01_PL1**-treated sample. (**d**) **HAP_R02_PL1**-treated sample.

Y132A mutation generates two binding sites per dimer for HAP molecules in the crystal structure reported here, while there is only one HAP1 site for two dimers from the low resolution capsid crystal structure^22^. Furthermore, it remains to be evaluated whether the HAP-bound Y132A structure is consistent with the interactions between HAP and misassembled capsid in solution. We defined the orientation of HAP upon binding to capsid assembly using a photoaffinity study coupled with liquid chromatography/tandem mass spectrometry (LC/MSMS). Three active photoaffinity probes containing an *R*-configuration at the 4^th^ position of the dihydropyrimidine ring were generated to map out the **HAP_R01**-interacting residues on core protein. Each of these probes possesses a photoreactive function group at different positions (6^th^, 5^th^, and 4^th^) of the dihydropyrimidine core of **HAP_R01**, named by **HAP_R01_PL1**, **HAP_R01_PL2**, and **HAP_R01_PL3**, respectively. It is well known that the corresponding 4^th^ *S*-diastereomer of **HAP** is less active than the 4^th^ *R*-diastereometer^13^,^23^. Thus the *S*-diastereomers were also synthesized as the negative controls, named by **HAP_R02_PL1**, **HAP_R02_PL2**, and **HAP_R02_PL3**, respectively (Fig. 4, Supplementary Figs S4 and S6). The *R*-configuration compounds retain reasonable activity in promoting capsid assembly and reducing viral DNA replication (Supplementary Table S2). A peak with molecular weight (Mw) increase of 477 Daltons was detected by LC/MS in samples treated with **HAP_R01_PL1**, but not in samples treated with **HAP_R02_PL1** (Fig. 4a,b). The intact mass increase exactly corresponds to the Mw of the carbene species derived from photo-activation (subtraction of 28 due to loss of two nitrogen atoms). **HAP_R01_PL2** and **HAP_R01_PL3** can also covalently label core protein with additional mass of 556 and 529 respectively. The labeling signals in the control **HAP_R02_PL2** and **HAP_R02_PL3**-treated samples are significantly lower than the active *R*-configured photolabels (Supplementary Figs S4 and S6).

To pin down the precise location of the photoprobe attachment, all six photolabel-treated samples were subject to proteolysis and LC/MSMS analysis. Different proteases, pepsin, protease K, chymotrypsin and trypsin, were applied separately to improve the coverage of fragmented ions to 100%. The MS2 data from three pairs of probes (**PL1**s, **PL2**s and **PL3**s) were searched for predicted peptide molecular weight increase. For two **PL1**s, photoreactive carbene derived from **HAP_R01_PL1** clearly forms an adduct with the pepsin-digested peptide 118–122 (**Y**LVSF). A series of A ions and B ions identify the labeling site at Y118 (Fig. 4c,d). For two **PL2**s, two MS2 spectra, 38–42 (**Y**RDAL) from protease K digestion and 29–39 (DLLDTAAAL**Y**R) from trypsin digestion, independently locate **HAP_R01_PL2** modification site at Y38 (Supplementary Fig. S5). For **PL3**s, weak B2 and B3 signals from chymotrypsin digested peptide 126–132 (**IR**TPPAY) narrow down the **HAP_R01_PL3** labeling site to N-terminus of the peptide. The modified residue is definitively pinpointed at R127 by multiple Y ions from different digestion runs, such as Y6 ion from the chymotryptic peptide 123–132 (GVWI**R**TPPAY) (Supplementary Fig. S7). Interestingly, both **HAP_R01_PL3** and **HAP_R02_PL3** are able to attach to Y88 in pepsin-digested peptides 84–94 (LVVS**Y**VNT) and 84–88 (LVVS**Y**), respectively (Supplementary Fig. S8). Since **HAP_R02_PL3** is an inactive control (Supplementary Table S2), we at first ruled out Y88 as a non-specific labeling site. However, a new space group was discovered during our crystallization screening of Y132A mutant. It may provide an explanation for the labeling of Y88 and a working model of core protein mis-assembly (see discussion).

The photo-incorporation of HAP derivatives enables us to map the labeling sites onto the capsid structure. The labeling sites Y118, Y38 and R127 are clustered at the dimer-dimer interface in capsid, consistent with the Y132A crystal structures. The pyrrolidine group at the 6-position of the HAP core is in the vicinity of Y118 from the concave; the 4^th^-positioned halogenated phenyl group is in direct contact with R127 from the cap; and methoxycarbonyl group at 5-position points towards Y38 (Fig. 3a). The flexible alkyl chain of **HAP_R01_PL2** allows the diazirine group to reach Y38, which is outside the binding concave.

### Prediction, confirmation and clinical relevance of binding site variants with susceptibility changes to HAP/SBA

Combining the crystal structures of core protein hexamer and the photoaffinity labeling sites on misassembled capsid, we find that the halogenated phenyl group of **HAP_R01** is in close contact with T33 of helix 2 and P25 of loop 2. We hypothesize that changing the T33 into an amino acid with a bulky sidechain would cause steric clash with the halogenated phenyl group, thereby reducing the inhibitory potency of **HAP_R01**. Indeed, this prediction can be verified by HBV DNA reduction measurement (Table 1). When mutating the T33 of core protein into N or Q, the mutant virus becomes less susceptible to **HAP_R01** inhibition, with a strong increase of the EC_50_ values of about 38- or 22-fold compared to the wild type virus, respectively. In comparison, replacing the T33 with S increases the EC_50_ only to 1.8 fold. The HAP compound **Bay 41-4109** shows a comparable pattern to **HAP_R01**. As the halogenated phenyl groups of **SBA_R01** and of **HAP_R01** are superimposable in the crystal structures (Fig. 3c), we expect a similar resistance pattern for the **SBA_R01** at position T33. The data again confirm our prediction: T33N and T33Q mutants show an over 13-fold change in EC_50_ by **SBA_R01**; in contrast, T33S mutant only shows a 2-fold increase. On the basis of the similar rationale, P25G mutant decreases the sensitivity to both SBA and HAP to different levels (12 to more than 67 fold EC_50_ increase). The glycine residue probably generates more space for the halogen-binding subpocket, and thus weakens the van der Waals contact with halogenated phenyl groups of HAP and SBA. Interestingly, P25A or P25S mutation leads to a significant decrease of HAP sensitivity in the range of 8- to 29-fold, whereas only a 2-fold reduction in sensitivity is observed when treated with **SBA_R01**. As the proline residue has unique dihedral angles, the mutations may lead to conformational changes of the loop 2 which contains key residues of the dimer interface like F23 (Fig. 3). We suspect the conformational rearrangement of loop2 might cause unfavourable interaction with the thiazole group of HAP. Because SBA does not possess the thiazole group, it may tolerate the conformation changes induced by P25A or P25S mutation. Finally, the V124I virus has increased susceptibility to all tested compounds. In contrast, changing the V124 to F increases the EC_50_ for the HAP compounds by 12- to 16-fold, whereas this mutation does not impact the sensitivity to **SBA_R01**. It appears that a small increase of the sidechain volume (V124I) favours the interaction with both **HAP_R01** and **SBA_R01**. A bulky substitution of V124F is likely causing steric clash with the 5^th^ positioned methoxycarbonyl group of HAP, but not with SBA (Fig. 3). As a control, all of the described mutants have little change in sensitivity to entecavir (**ETV)** (Table 1, Supplementary Fig. S9). Replication capacity of mutant viruses varies from 6% to 99% compared to WT virus (Supplementary Fig. S10). The evidence from cell-based assay confirms our prediction on mutants with altered HAP/SBA potency, thus indicating that the compound binding pocket at the dimer-dimer interface observed in the Cp149_Y132A structure resembles the HAP interacting site in capsid.

The prevalence of these amino acid changes in HBV from infected patients was evaluated using an internal surveillance database where 3953 core protein sequences from HBV infected samples deposited in NCBI were selected via text-mining. The results show T33N and T33S found in 0.075% and 0.05% of the cases respectively (Table 2). Given the quasispecies nature of HBV, we also evaluated the prevalence of those amino acid substitutions at low level within samples from treatment-naïve patients. In this exploratory study, the HBV genomes from 50 chronically HBV-infected treatment-naïve Chinese patients were subjected to ultra-deep pyrosequencing (UDPS)^29^. Interestingly, we find low-abundance quasispecies (frequency bellow 2.5%) containing substitutions P25S in one patient and V124F in four patients (Table 2).

## Discussion

**Figure 5:**
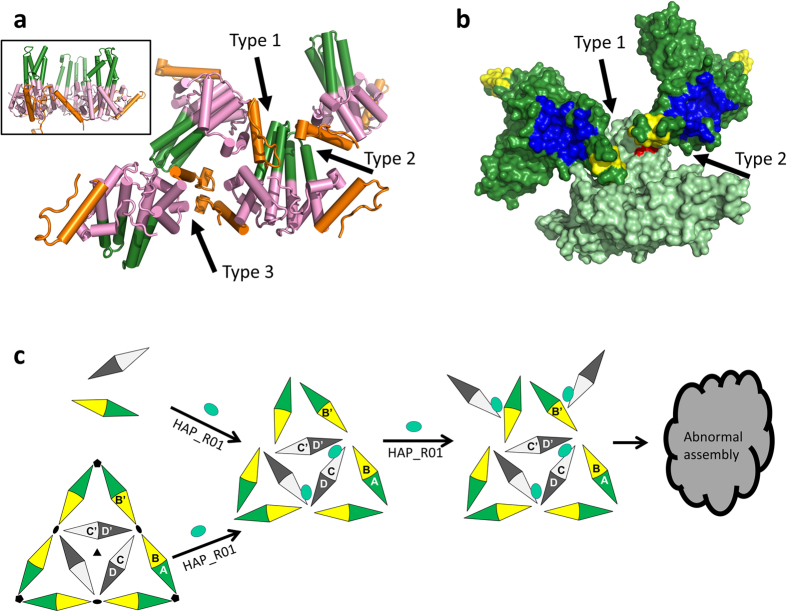
The novel crystal packing suggests a working model of HAP-induced mis-assembly. (**a**) Packing of tetragonal (Space Group: P41212) Y132A crystal showing three different types of interfaces. The spike tip (G63-G94) is colored in green. The intersubunit contact domain (G111-C-terminus) is represented in orange. Black arrows indicate type 1 (spike-to-contact), type 2 (contact-to-spike) and type 3 (contact-to-contact) interfaces, respectively. Inlet represents a typical side-by-side packing from P1 crystals. (**b**) Surface representation of type 1 and type 2 interfaces. Y132A structure is colored in light green. Two neighboring symmetry mates are colored in dark green. The concave for HAP binding on the symmetry mates are colored in blue. The cap for HAP binding on the symmetry mates is colored in yellow. Red surface highlights the photoaffinity labeling site Y88 by **HAP_R01_PL3** and **HAP_R02_PL3**, which is close to the cap in this new crystal packing. The spike tip and cap may create a potential pocket to bind HAP. (**c**) HAP can accelerate dimer assembly and disrupt preformed capsid. HAP predominantly binds to dimer interface and enhances assembly kinetics at low concentration. With excess of HAP, new contact formats are created as HAP perturbs the thermal dynamics of normal assembly.

New generation capsid assembly modulators are currently under clinical investigations^12^. Structural information on different classes of capsid inhibitors will greatly facilitate the rationale design of drug candidates. The unexpected difference between HAP1^22^ and HAP18^30^ bound structures suggests that compounds of the same HAP series may have distinct thermodynamic consequences on capsid. Thus, understanding the mechanism of each clinical candidate is warranted. It is technically challenging to obtain complex structures of HBV capsid with inhibitors at a reasonable resolution to unambiguously define the molecular interactions. In addition, at equal molar concentration of core protein dimer, **HAP_R01** induces heterogeneous structures of core protein (Fig. 1d). The full occupancy of **HAP_R01** and homogeneity of capsid become two conflicting factors to prevent us from obtaining the capsid-**HAP_R01** complex structure. We used the core protein Y132A mutant to trap the oligomer of dimers. During the crystallization screening, a new space group P41212 was identified, where core protein dimers pack in largely different formats from open triangles (3KXS)^27^ and closed triangles (4BMG)^28^. The packing in the new crystal lattice, termed spike-contact packing, generates three types of interfaces. The type 1 (spike-to-contact) and type 2 (contact-to-spike) interfaces are formed by the spike domain (G63-G94) in one dimer and the intersubunit contact domain (G111-C terminus) from the symmetry-related dimer, with a contact surface area of 856.4 Å^2^ and 414.8 Å^2^, respectively. The type 3 interface (contact-to-contact) is constructed by the contact domains from two neighbouring dimers packing in an upside-down manner with 332.3 Å^2^ contact area (Fig. 5a). The contact areas are in similar order to the closed triangle format (444.4 to 484.9 Å^2^ in Y132A-**HAP_R01** structure). The novel apo structure suggests that at high local concentration, core protein dimer has a tendency to associate with each other in different formats, not limited to triangle format. Interestingly, during our photoaffinity study, we identified Y88 in the spike region as an additional label site for both *R*- and *S*-configuration HAPs (Supplementary Fig. S8). In the tetragonal crystal, Y88 is in the vicinity of the compound-binding cap from symmetry-related dimers (Fig. 5b). Especially in the type 2 interface, the pocket may accommodate either of the HAP stereoisomers (Supplementary Fig. S11). As core protein modulators may trap capsid in different states^22^,^30^,^31^, the spike-contact format or upside-down format of core protein packing may represent one state of the total ensembles. Based on these observations, we propose a working model of HAP in misdirecting core protein assembly. At sub-stoichiometric concentration, **HAP_R01** preferentially binds to dimer interface and accelerates assembly as the contact domain interacts with the contact domain of the other dimers. With the increase of **HAP_R01** concentration, the probability of the contact domain associating with spike domain or with the contact domain in upside-down format is greatly enhanced. With small mistakes guided by **HAP_R01** during the assembly process, the perfect icosahedron cannot be produced (Fig. 5c).

Biochemical and biophysical evidence has shown that **SBA_R01** and **HAP_R01** have drastically different effects on core assembly. We hypothesize that **HAP_R01** can perturb the dimer-dimer interface, thereby changing the nucleation (presumably hexamer) pattern and increasing the probability of recruiting spike-to-contact dimers to the nucleation center. Although **SBA_R01** binds to the same interface, the conformational changes may be so small that the energetic difference is not sensed by other dimers. We performed energy minimizations of **HAP_R01** and **SBA_R01** at the C-D’ interface of WT capsid. Only **HAP_R01**, but not **SBA_R01**, induces significant conformational changes at the C-terminus of the helix 5 (S121-P129) on chain D’ (Supplementary Fig. S12). The distortion of the helix 5 may cause steric clash with Y132 and A131 of chain B’, which is related to chain B by the two-fold symmetry. As mentioned above, Y132 is critical for capsid assembly. Rearrangement at this location may disturb the perfect icosahedral architecture. In contrast to the relatively flexible amide bond of **SBA_R01**, the rigid dihydropyrimidine moiety of **HAP_R01**, together with the extra thiazole group may be responsible for the allosteric effects. It is conceivable that the small distortion may be amplified in the process of the multiple dimer association^32^. The chance of incomplete or abnormal core protein association is greatly enhanced (Fig. 5).

Different HAP molecules misdirect core protein into various products, such as heterogeneous pleiomorphic structures or regular long tubes^21^. Thus, the non-capsid polymer induced by the new HAP molecule **HAP_R01** is worthy of investigation. The aberrant assembly caused by **HAP_R01** may trigger its depletion, probably via proteasome degradation pathway, similar to **Bay 41-4109**^13^. The inhibitory effect of core protein depletion on HBV replication is striking as capsid is essential for viral DNA synthesis. In comparison, **SBA_R01** does not lead to core protein reduction. The core protein turn-over may not be significantly affected by SBA. The consequence of this mechanistic difference among capsid inhibitors remains to be further investigated as core protein is highly immunogenic in chronic infections and the epitopes in the spike region on the shell of capsid is optimal for cross-linking B cell receptor leading to upregulation of costimulatory factors^33^,^34^,^35^

With the achievement of clinical cure for HIV and HCV by combination therapies, HBV has become one of the focused areas of antiviral research. Unlike HIV and HCV, currently there is no reliable cell-based re-infection system to evaluate the fitness of designed mutations. The resistance development in patients upon chronic HAP or SBA treatment remains elusive. Any prediction tool prior to drug treatment is potentially of great clinical value. With the predictive power of the crystal structures and the application of deep sequencing, we identified the natural existence of core protein variants in treatment-naïve patients that may exhibit different sensitivity to HAP and SBA. Although it is unclear whether these mutants would survive during the treatment of capsid inhibitors, we could predict that the HAP-resistant mutant V124F could be suppressed by **SBA_R01**.

In summary, our study has elucidated the distinctive molecular mechanisms of HAP and SBA series that target on HBV capsid for the first time, although they are hitting on the similar dimer-dimer interface. The detailed interaction map and the novel action model can accelerate drug development against this important viral target. The predictive power relied on crystal structures and photoaffinity labeling of capsid assembly enables us to scrutinize the development of drug resistance and design the right molecule to counter the potential resistance.

## Methods

### Protein expression and purification

C-terminal 6XHis-tagged Cp149_Y132A from HBV genotype D strain adyw was constructed as described previously^28^ and cloned into pET21a vector. Detailed expression and purification with modification is provided in Supplementary Information.

### Capsid assembly fluorescence quenching assay

Cp150 was purified and labeled with maleimidyl BoDIPY-FL dye (Sigma) as previously described^36^. Detailed information is provided in Supplementary Information.

### HepG2.2.15 anti-HBV assay

The extracellular HBV DNA from HepG2.2.15 cell culture was quantified by real time PCR. The cytotoxicity was measured using CCK-8 kit (Dongjindo). See Supplementary Information for details.

### Particle gel assay

HBV capsids and encapsidated DNA were analyzed by a native agarose gel electrophoresis-based assay. Briefly, HepG2.2.15 cells cultured in 12-well plates were lysed by the addition of 300 μL of buffer containing 10 mM Tris-HCl (pH 7.4), 100 mM NaCl, 1 mM EDTA, and 0.5% CA-630 per well. Cell debris was removed by centrifugation at 6,000 rpm for 5 mins. The viral particles were fractionated by electrophoresis through nondenaturing 1.2% agarose gels and capillarilly transferred to a nitrocellulose filter by blotting with 20X SSC buffer (Thermo Fisher). HBV capsids were detected by probing the membrane with an antibody against HBV core protein (Dako). Bound antibodies were revealed by horseradish peroxidase (HRP)-labeled secondary antibodies (Sigma) and visualized with Pierce™ ECL Western Blotting Substrate (Thermo Fisher).

To detect encapsidated HBV DNA, the viral particles were also fractionated by electrophoresis through nondenaturing 1.2% agarose gels. The gel was treated with a denaturing solution containing 0.5 M NaOH and 1.5 M NaCl for 30 min, followed by neutralization with a buffer containing 1 M Tris-HCl pH 7.4 and 1.5 M NaCl for 30 min. DNA was then transferred to a Hybond-XL membrane (GE Healthcare) in 20 X SSC buffer, and detected by Southern blot hybridization with a DIG-labeled HBV probe.

HBV core protein was detected by standard Western blot analysis. Denatured cell lysate was resolved in a NuPAGE^®^ 4–12% Bis Tris gel (Thermo Fisher Scientific), and protein was transferred to a Hybond-ECL membrane (GE Healthcare). The membrane was probed with antibodies against HBV core protein (Dako) and β-actin (Sigma). Bound antibodies were revealed by horseradish peroxidase (HRP)-labeled secondary antibodies (Sigma) and visualized with Pierce™ ECL Western Blotting Substrate.

### Electron Microscopy

5 μM Cp150 dimer was mixed with either 5 μM or 20 μM compound in a buffer containing 250 mM NaCl, 50 mM HEPES (pH7.4) and 0.2% DMSO. The solution was incubated at 37 °C for 2 hours prior to negative staining. Samples were adsorbed to freshly glow discharged carbon coated grids, and stained in fresh 1% (wt/vol) phosphotungstic acid for 10 s. After absorbing excess water by filter paper and drying, the grids were examined with a JEM-2100 transmission electron microscope operating at 200 kV. Images were typically acquired with a magnification of 40,000x.

### Crystallization and structure determination

17 mg/ml protein of Cp149_Y132A in 20 mM Tris buffer (pH 9.0) and 2 mM DTT was combined with equal volume of crystallization buffer containing 100 mM citrate (pH 5.6), 21% (vol/vol) isopropanol, and 1% (W/V) PEG 10,000. The best apo crystal (0.17 mm × 0.46 mm) appeared almost a month later after setting up the sitting drop on MRC2 microplates at 20 °C. Apo crystal was soaked with 2 mM compound in the crystallization buffer plus 25% glycerol for 2 hours, then was picked out with cryoloop and flash frozen in liquid nitrogen.

Cp149_Y132A crystal in P41212 form grew in the well buffer containing 5.4 M ammonium nitrate and 0.1 M Bis-tris propane pH 7.0, within 2 days after setting up the sitting drop at 11.3 mg/ml. 30% glucose in the original well solution was used as cryoprotectant and crystals were flash frozen in liquid nitrogen. The datasets were collected on single crystals for Y132A-**HAP_R01** or Y132A-**SBA_R01** complex structure at BL17U beamline, Shanghai Synchrotron Radiation Facility (SSRF). Two crystals were averaged for apo Y132A crystal in P41212 space group. Data were processed using HKL2000^37^. Molecular replacement solution was determined by PHASER^38^ using 4BMG as the search model. The structure was refined and manually built several rounds using phenix.refine^39^ and COOT^40^. The final model quality was evaluated by MolProblity^41^ of the PHENIX package^42^ (Supplementary Table S1).

### Rational design and synthesis of photoreactive HAPs

Diazirine group was incorporated into 6^th^ or 5^th^ position at the dihydropyrimidine core of **HAP_R01** to generate photoaffinity label **HAP_R01_PL1** or **HAP_R01_PL2**, respectively. Azido group was incorporated into 4^th^ position of **HAP_R01** to generate photoaffinity label **HAP_R01_PL3**. The corresponding inactive diastereoisomers were also synthesized and named **HAP_R02_PL1**, **HAP_R02_PL2**, and **HAP_R02_PL3**, respectively. The detailed procedures and characterization of these compounds are disclosed in the Supplementary Information.

### Photoaffinity labeling and mass spectrometry

The detailed methods for photoaffinity labeling, protease digestion, LC/MS and LC/MSMS analysis can be found in Supplementary Information. The mass spec data files were processed using Analyst TF 1.6 to extract the mgf files. The resulting mgf files were used to search a customized database containing the sequences of Cp150 (MASCOT). The search was conducted with corresponding enzyme specificity and a HAP compound (exact mass of 476.07, 554.10 or 527.06 for **PL1**, **PL2** or **PL3** respectively) at every amino acid as the variable modification. The mass tolerance for parent ion and fragment ion was set at 0.1 Da. The database searching results were further validated manually to determine the photo-labeled peptide identity.

### HBV transient transfection assay

An HBV wildtype plasmid was created by insertion of a 1.3x genotype D HBV genome (NCBI accession: V01460.1) into the pBR322 vector. HBV core variants, T33/N/Q/S, P25A/G/S, and V124F/I, were generated by site directed mutagenesis PCR. Sequencing of the whole length HBV genomes confirmed that only the intended nucleotide changes were present in the final constructs.

HepG2 cells (ATCC) cultured in DMEM/F12 medium (supplemented with 10% FBS, 1% Pen/Strep and 1% MEM NEAA) were transfected with HBV plasmids using X-tremeGENE HP DNA Transfection Reagent (Roche Diagnostics) according to the manufacturer’s protocol, seeded into 96-well plates (20,000 cells and 50 ng HBV plasmid per well), and incubated over night at 37 °C and 5% CO_2_. Transfection mixtures were removed the following day, and cells were treated with serially diluted compounds for seven days at a final DMSO concentration of 1%, after which intracellular encapsidated HBV DNA was extracted from cells. Briefly, transfected HepG2 cells were lysed for 10 mins at room temperature using 0.5% CA-630 buffer (10 mM Tris HCl, pH 8.0, 0.5% CA-630,150 mM NaCl), centrifuged for 10 mins at 4 °C, and supernatants were treated with DNase I for 2 h at 37 °C to remove plasmid DNA before reaction was stopped using EDTA. Supernatants were centrifuged and encapsidated HBV DNA was isolated using QuickExtract Solution (Epicentre Biotechnologies) according to the manufacturer’s protocol. Encapsidated HBV DNA was measured by qPCR performed on a Light Cycler 480 (Roche) using the Light Cycler 480 Probe Master (Roche) and the following TaqMan HBV primers and probe (Shanghai ShineGene):

Forward primer: AAG AAA AAC CCC GCC TGT AA

Reverse primer: CCT GTT CTG ACT ACT GCC TCT CC

Probe: 5′+TARMA+CCT GAT GTG ATG TTC TCC ATG TTC AGC+BHQ2–3′.

DNA copy numbers were calculated from C_t_ values based on a HBV plasmid DNA standard curve by the Light Cycler 480 software. Percent inhibition values for drug treated samples were calculated by normalizing to values from HepG2 cells only treated with DMSO. EC_50_ values were determined from the percent inhibition data obtained at different compound concentrations by non-linear fitting using GraphPad Prism 6 software. EC_50_ fold-change values (FC) were calculated by dividing the mutant EC_50_ value by the wild type EC_50_ value. To determine the replication capacity of HBV core variants, the values for HBV DNA from the qPCR assay obtained from cells transfected with HBV variants were compared with those obtained from the wild type HBV transfection.

## Additional Information

**Accession codes:** Atomic coordinates and structure factors of Apo core protein mutant Y132A, Y132A-**SBA_R01** and Y132A-**HAP_R01** have been deposited in the Protein Data Bank (http://www.pdb.org) with the accession codes 5WTW, 5T2P and 5WRE respectively.

**How to cite this article:** Zhou, Z. *et al*. Heteroaryldihydropyrimidine (HAP) and Sulfamoylbenzamide (SBA) Inhibit Hepatitis B Virus Replication by Different Molecular Mechanisms. *Sci. Rep.* **7**, 42374; doi: 10.1038/srep42374 (2017).

**Publisher's note:** Springer Nature remains neutral with regard to jurisdictional claims in published maps and institutional affiliations.

## Supplementary Material

Supplementary Information

## Figures and Tables

**Table 1:** Antiviral activity of HAP or SBA against HBV core mutants determined in the HepG2 HBV transient transfection assay.

	HAP_R01		Bay 41-4109		SBA_R01		ETV	
	Mean EC_50_ (nM) ± SD	FC	Mean EC_50_ (nM) ± SD	FC	Mean EC_50_ (nM) ± SD	FC	Mean EC_50_ (nM) ± SD	FC
WT	91 ± 10	1	149 ± 25	1	390 ± 44	1	1.40 ± 0.20	1
Core T33N	3471 ± 2065	38.1	>10000	>67	>5000	>13	1.16 ± 0.01	0.8
Core T33Q	1972 ± 607	21.7	3722 ± 890	25.0	>5000	>13	2.09 ± 0.73	1.5
Core T33S	162 ± 46	1.8	451 ± 205	3.0	878 ± 112	2.3	1.35 ± 0.25	1.0
Core P25G	1384 ± 62	15.2	>10000	>67	4749 ± 337	12.2	2.23 ± 0.52	1.6
Core P25A	735 ± 118	8.1	3452 ± 1438	23.2	895 ± 71	2.3	1.34 ± 0.12	1.0
Core P25S	902 ± 86	9.9	4278 ± 461	28.7	930 ± 168	2.4	1.10 ± 0.46	0.8
Core V124I	9 ± 1	0.1	13 ± 3	0.09	42 ± 2	0.1	0.69 ± 0.25	0.5
Core V124F	1086 ± 113	11.9	2332 ± 799	15.7	289 ± 102	0.7	2.53 ± 0.68	1.8

Shown are mean EC_50_ values ± standard deviation (SD) from three independent experiments. EC_50_ fold-change values (FC) were calculated by dividing the mutant EC_50_ value by the wild type EC_50_ value.

**Table 2:** Mutation occurrence in HBV infected treatment-naïve patients.

	NCBI	UDPS	
	Prevalence	Prevalence	Intra-host frequency
Core T33N	3/3953 (0.075%)	0/50 (0%)	—
Core T33S	2/3953 (0.05%)	0/50 (0%)	—
Core P25S	0/3953 (0%)	1/50 (2%)	2.47%
Core V124F	0/3953 (0%)	4/50 (8%)	1.12%, 1.46%, 2.06%, 2.08%

Prevalence of the core protein variants at selected sites from NCBI database and from UDPS results is listed here. 3953 sequences from NCBI were analyzed. UDPS was performed on HBV samples from 50 chronically HBV infected treatment-naïve Chinese patients. Intra-host frequency in UDPS denotes the abundance of the mutations within the sample viral population. The detection limit for UDPS is 0.05%.

## References

[b1] World Health Organization, Hepatitis B: World Health Organization Fact Sheet 204. http://www.who.int/mediacentre/factsheets/fs204/en/ (Date of access: 13/09/2016) (2016).

[b2] Chen D. S. Hepatitis B vaccination: The key towards elimination and eradication of hepatitis B. J Hepatol 50, 805–816, doi: 10.1016/j.jhep.2009.01.002 (2009). 19231008

[b3] Kwon H. & Lok A. S. Hepatitis B therapy. Nat Rev Gastroenterol Hepatol 8, 275–284, doi: 10.1038/nrgastro.2011.33 (2011). 21423260

[b4] Hu Y. . Novel Therapeutics in Discovery and Development for Treatment of Chronic HBV Infection. 48, 265–281, doi: 10.1016/b978-0-12-417150-3.00017-x (2013).

[b5] Ganem D. & Varmus H. E. The molecular biology of the hepatitis B viruses. Annu Rev Biochem 56, 651–693, doi: 10.1146/annurev.bi.56.070187.003251 (1987). 3039907

[b6] Chisari F. V. & Ferrari C. Hepatitis B virus immunopathogenesis. Annu Rev Immunol 13, 29–60, doi: 10.1146/annurev.iy.13.040195.000333 (1995). 7612225

[b7] Crowther R. A. . Three-dimensional structure of hepatitis B virus core particles determined by electron cryomicroscopy. Cell 77, 943–950 (1994). 8004680 10.1016/0092-8674(94)90142-2

[b8] Wynne S. A., Crowther R. A. & Leslie A. G. The crystal structure of the human hepatitis B virus capsid. Mol Cell 3, 771–780 (1999). 10394365 10.1016/s1097-2765(01)80009-5

[b9] Bock C. T. . Structural organization of the hepatitis B virus minichromosome. J Mol Biol 307, 183–196, doi: 10.1006/jmbi.2000.4481 (2001). 11243813

[b10] Guo Y. H., Li Y. N., Zhao J. R., Zhang J. & Yan Z. HBc binds to the CpG islands of HBV cccDNA and promotes an epigenetic permissive state. Epigenetics 6, 720–726 (2011). 21546797 10.4161/epi.6.6.15815

[b11] Gruffaz M. . The nuclear function of Hepatitis B capsid (HBc) protein is to inhibit IFN response very early after infection of hepatocytes. Hepatology 58, 276a–276a (2013).

[b12] Durantel D. & Zoulim F. New antiviral targets for innovative treatment concepts for hepatitis B virus and hepatitis delta virus. J Hepatol 64, S117–131, doi: 10.1016/j.jhep.2016.02.016 (2016). 27084032

[b13] Deres K. . Inhibition of hepatitis B virus replication by drug-induced depletion of nucleocapsids. Science 299, 893–896, doi: 10.1126/science.1077215 (2003). 12574631

[b14] Feld J. J. . The phenylpropenamide derivative AT-130 blocks HBV replication at the level of viral RNA packaging. Antiviral Res 76, 168–177, doi: 10.1016/j.antiviral.2007.06.014 (2007). 17709147

[b15] Wu G. . Preclinical characterization of GLS4, an inhibitor of hepatitis B virus core particle assembly. Antimicrob Agents Chemother 57, 5344–5354, doi: 10.1128/AAC.01091-13 (2013). 23959305 PMC3811253

[b16] Wang Y. J. . A novel pyridazinone derivative inhibits hepatitis B virus replication by inducing genome-free capsid formation. Antimicrob Agents Chemother 59, 7061–7072, doi: 10.1128/AAC.01558-15 (2015). 26349829 PMC4604411

[b17] Zlotnick A. . Core protein: A pleiotropic keystone in the HBV lifecycle. Antiviral Res 121, 82–93, doi: 10.1016/j.antiviral.2015.06.020 (2015). 26129969 PMC4537649

[b18] Weber O. . Inhibition of human hepatitis B virus (HBV) by a novel non-nucleosidic compound in a transgenic mouse model. Antiviral Res 54, 69–78 (2002). 12062392 10.1016/s0166-3542(01)00216-9

[b19] Stray S. J. & Zlotnick A. BAY 41-4109 has multiple effects on Hepatitis B virus capsid assembly. J Mol Recognit 19, 542–548, doi: 10.1002/jmr.801 (2006). 17006877

[b20] Stray S. J. . A heteroaryldihydropyrimidine activates and can misdirect hepatitis B virus capsid assembly. Proc Natl Acad Sci USA 102, 8138–8143, doi: 10.1073/pnas.0409732102 (2005). 15928089 PMC1149411

[b21] Bourne C. . Small-molecule effectors of hepatitis B virus capsid assembly give insight into virus life cycle. J Virol 82, 10262–10270, doi: 10.1128/JVI.01360-08 (2008). 18684823 PMC2566253

[b22] Bourne C. R., Finn M. G. & Zlotnick A. Global structural changes in hepatitis B virus capsids induced by the assembly effector HAP1. J Virol 80, 11055–11061, doi: 10.1128/JVI.00933-06 (2006). 16943288 PMC1642186

[b23] Klumpp K. . High-resolution crystal structure of a hepatitis B virus replication inhibitor bound to the viral core protein. Proc Natl Acad Sci USA 112, 15196–15201, doi: 10.1073/pnas.1513803112 (2015). 26598693 PMC4679053

[b24] Qiu Z. . Design and Synthesis of Orally Bioavailable 4-Methyl Heteroaryldihydropyrimidine Based Hepatitis B Virus (HBV) Capsid Inhibitors. J Med Chem, doi: 10.1021/acs.jmedchem.6b00879 (2016). 27458651

[b25] Campagna M. R. . Sulfamoylbenzamide derivatives inhibit the assembly of hepatitis B virus nucleocapsids. J Virol 87, 6931–6942, doi: 10.1128/JVI.00582-13 (2013). 23576513 PMC3676120

[b26] Bourne C. R., Katen S. P., Fulz M. R., Packianathan C. & Zlotnick A. A mutant hepatitis B virus core protein mimics inhibitors of icosahedral capsid self-assembly. Biochemistry 48, 1736–1742, doi: 10.1021/bi801814y (2009). 19196007 PMC2880625

[b27] Packianathan C., Katen S. P., Dann C. E. 3rd & Zlotnick A. Conformational changes in the hepatitis B virus core protein are consistent with a role for allostery in virus assembly. J Virol 84, 1607–1615, doi: 10.1128/JVI.02033-09 (2010). 19939922 PMC2812345

[b28] Alexander C. G. . Thermodynamic origins of protein folding, allostery, and capsid formation in the human hepatitis B virus core protein. Proc Natl Acad Sci USA 110, E2782–2791, doi: 10.1073/pnas.1308846110 (2013). 23824290 PMC3725087

[b29] Su C. . Association study between mannose-binding lectin haplotypes and X gene mutation of hepatitis B virus from treatment naive patients. Aging 8, 2862–2870, doi: 10.18632/aging.101097 (2016). 27824315 PMC5191875

[b30] Venkatakrishnan B. . Hepatitis B Virus Capsids Have Diverse Structural Responses to Small-Molecule Ligands Bound to the Heteroaryldihydropyrimidine Pocket. J Virol 90, 3994–4004, doi: 10.1128/JVI.03058-15 (2016). 26842475 PMC4810570

[b31] Katen S. P., Tan Z., Chirapu S. R., Finn M. G. & Zlotnick A. Assembly-directed antivirals differentially bind quasiequivalent pockets to modify hepatitis B virus capsid tertiary and quaternary structure. Structure 21, 1406–1416, doi: 10.1016/j.str.2013.06.013 (2013). 23871485 PMC3756818

[b32] Prevelige P. E. Jr. Inhibiting virus-capsid assembly by altering the polymerisation pathway. Trends Biotechnol 16, 61–65 (1998). 9487732 10.1016/s0167-7799(97)01154-2

[b33] Bottcher B., Wynne S. A. & Crowther R. A. Determination of the fold of the core protein of hepatitis B virus by electron cryomicroscopy. Nature 386, 88–91, doi: 10.1038/386088a0 (1997). 9052786

[b34] Conway J. F. . Visualization of a 4-helix bundle in the hepatitis B virus capsid by cryo-electron microscopy. Nature 386, 91–94, doi: 10.1038/386091a0 (1997). 9052787

[b35] Milich D. R. . Role of B cells in antigen presentation of the hepatitis B core. Proc Natl Acad Sci USA 94, 14648–14653 (1997). 9405667 10.1073/pnas.94.26.14648 PMC25082

[b36] Zlotnick A. . *In vitro* screening for molecules that affect virus capsid assembly (and other protein association reactions). Nat Protoc 2, 490–498 (2007). 17406612 10.1038/nprot.2007.60 PMC2099249

[b37] Otwinowski Z. & Minor W. Processing of X-ray diffraction data collected in oscillation mode. Method Enzymol 276, 307–326, doi: 10.1016/S0076-6879(97)76066-X (1997). 27754618

[b38] McCoy A. J. . Phaser crystallographic software. J Appl Crystallogr 40, 658–674, doi: 10.1107/S0021889807021206 (2007). 19461840 PMC2483472

[b39] Afonine P. V. . Towards automated crystallographic structure refinement with phenix.refine. Acta Crystallogr D Biol Crystallogr 68, 352–367, doi: 10.1107/S0907444912001308 (2012). 22505256 PMC3322595

[b40] Emsley P. & Cowtan K. Coot: model-building tools for molecular graphics. Acta Crystallogr D Biol Crystallogr 60, 2126–2132, doi: 10.1107/S0907444904019158 (2004). 15572765

[b41] Chen V. B. . MolProbity: all-atom structure validation for macromolecular crystallography. Acta Crystallogr D Biol Crystallogr 66, 12–21, doi: 10.1107/S0907444909042073 (2010). 20057044 PMC2803126

[b42] Adams P. D. . PHENIX: a comprehensive Python-based system for macromolecular structure solution. Acta Crystallogr D Biol Crystallogr 66, 213–221, doi: 10.1107/S0907444909052925 (2010). 20124702 PMC2815670

