# Supplementary material for: Heteroaryldihydropyrimidine (HAP) and Sulfamoylbenzamide (SBA) Inhibit Hepatitis B Virus Replication by Different Molecular Mechanisms
Source: Sci Rep. 2017 Feb 13;7:42374. doi: 10.1038/srep42374 (PMC5304331; doi:10.1038/srep42374)
Supplement: Supplementary Information [file srep42374-s1.pdf]

Heteroaryldihydropyrimidine (HAP) and sulfamoylbenzamide (SBA) Inhibit Hepatitis B Virus Replication by Different Molecular Mechanisms

Zheng Zhou[1,*], Taishan Hu[2], Xue Zhou[3], Steffen Wildum[3,4], Fernando Garcia-Alcalde[3,4], Zhiheng Xu[1], Daitze Wu[3], Yi Mao[3], Xiaojun Tian[3], Yuan Zhou[3], Fang Shen[3], Zhisen Zhang[2], Guozhi Tang[2], Isabel Najera[3,4], Guang Yang[3], Hong C. Shen[2], John A.T. Young[3,4], and Ning Qin[1]

Roche Pharma Research and Early Development, [1]Chemical Biology, [2]Medicinal Chemistry, [3]Immunology, Inflammation and Infectious Diseases Discovery and Translational Area, [4]Roche Innovation Center Basel, Grenzacherstrasse 124, CH-4070 Basel, Switzerland; Roche Innovation Center Shanghai, 720 Cailun Road, Shanghai, 201203 China

Corresponding Author
*E-mail: dr_zhengzhou@hotmail.com
Telephone: +86-21-28946778
Fax: +86-21-50790292

Supplementary Figures 2
Supplementary Tables 15
Supplementary Methods 17
Supplementary References 27

Supplementary Figures

Supplementary Figure S1.

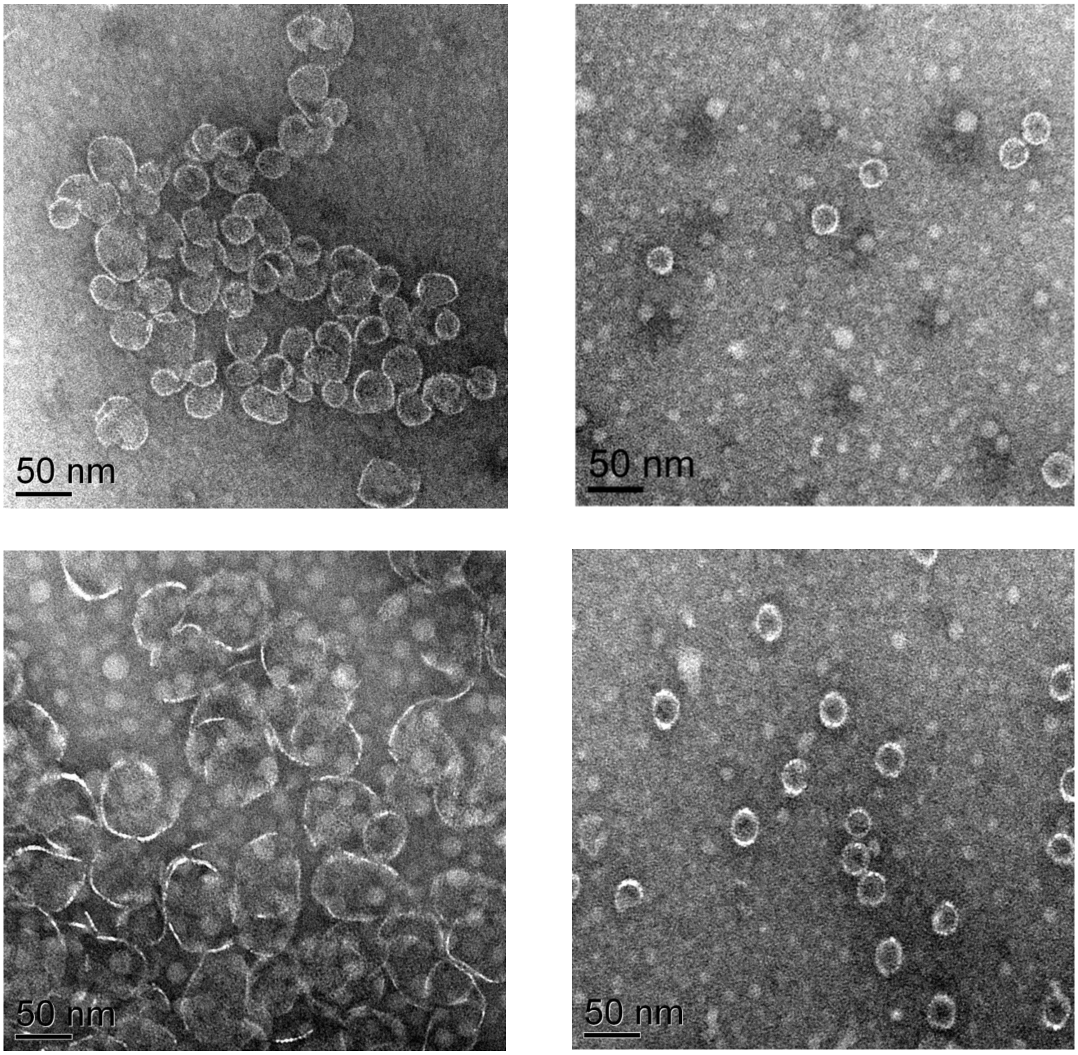

Supplementary Figure S1. Electron micrographs of compound-treated core protein assembly at different dimer-to-compound ratios. Up left: 5 μM core protein dimer incubated with 5 μM **HAP_R01**. The same image in Figure 1d is used for comparison. Up right: 5 μM core protein dimer incubated with 5 μM **SBA_R01**. The same image in Figure 1d is used for comparison. Bottom left: 5 μM core protein dimer incubated with 20 μM **HAP_R01**. Bottom right: 5 μM core protein dimer incubated with 20 μM **SBA_R01**. Black scale bar indicates 50 nm.

Supplementary Figure S2.

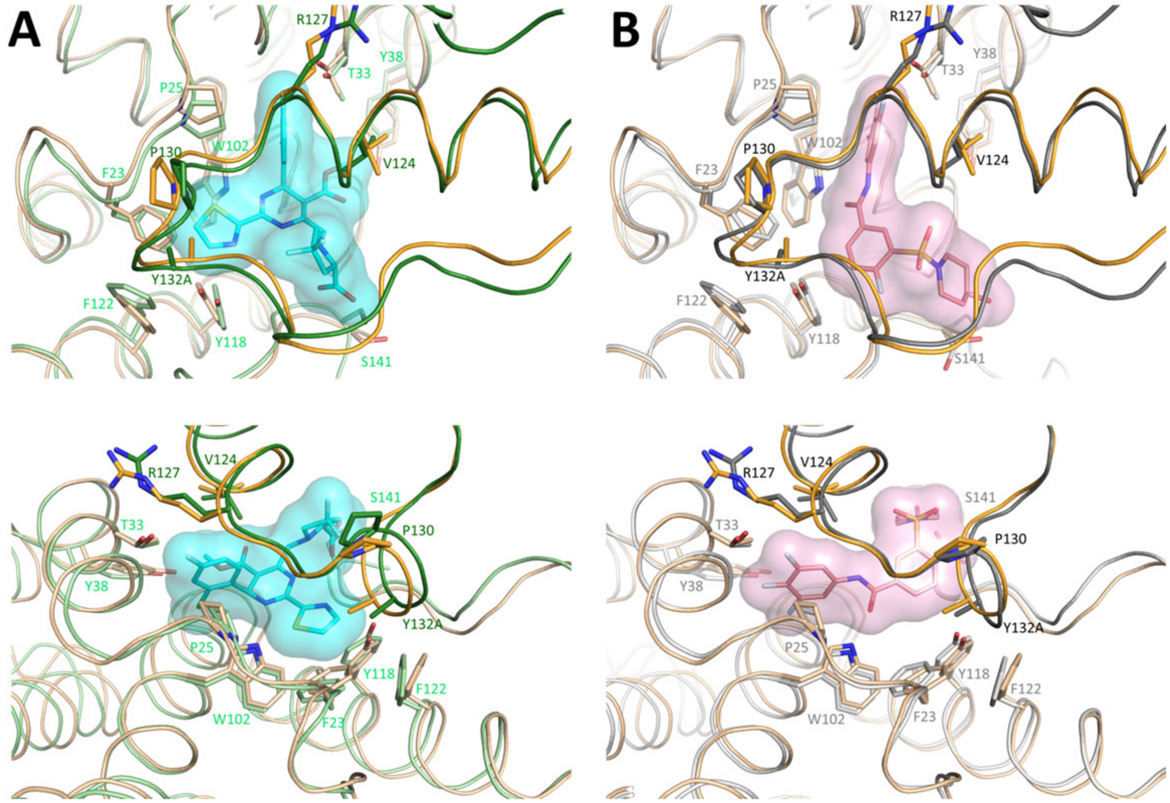

Supplementary Figure S2. Binding sites of **HAP_R01** and **SBA_R01**. The compound binding pocket at B-C interface is shown as an example. (A, B) Overlay of apo Y132A structure (4BMG) with **HAP_R01** bound (A) or **SBA_R01** bound structure (B). Chain B and Chain C of apo Y132A structure are colored in light orange and dark orange respectively. The color schemes of Y132A-**HAP_R01** and Y132A-**SBA_R01** structures are the same as Fig. 3. Upper panel: top view from spike to contact domain. Lower panel: selected side view for clear representation of ligand-protein interactions.

Supplementary Figure S3.

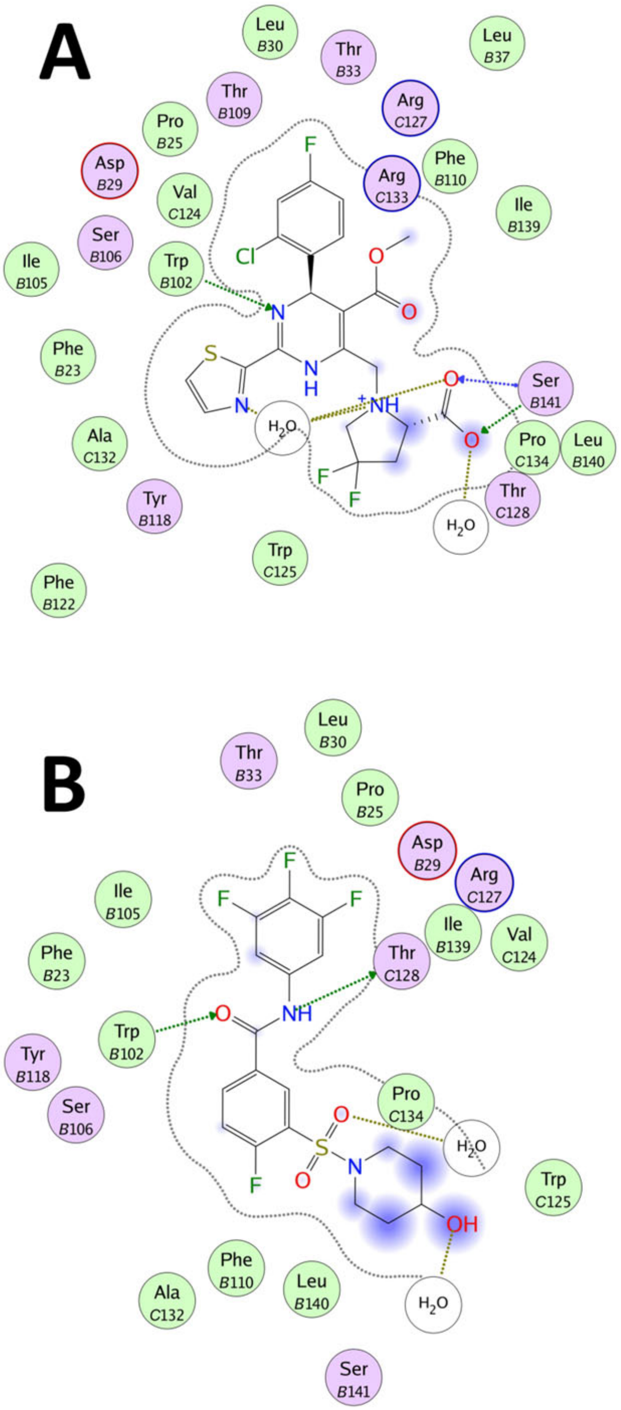

Supplementary Figure S3. 2D diagrams of ligand interactions. Dotted boundary indicates the proximity contour of the ligand. Green and pink circles represent hydrophobic and polar residues within 4.5 Å cut-off distance, respectively. Blue dots represent exposed atoms in ligands. Interactions with main chain atoms or side chain atoms of core protein or water are shown in blue or green or brown dotted lines, respectively. Arrowheads point towards the hydrogen bond acceptor. (A) **HAP_R01**-core protein interaction. (B) **SBA_R01**-core protein interaction.

Supplementary Figure S4.

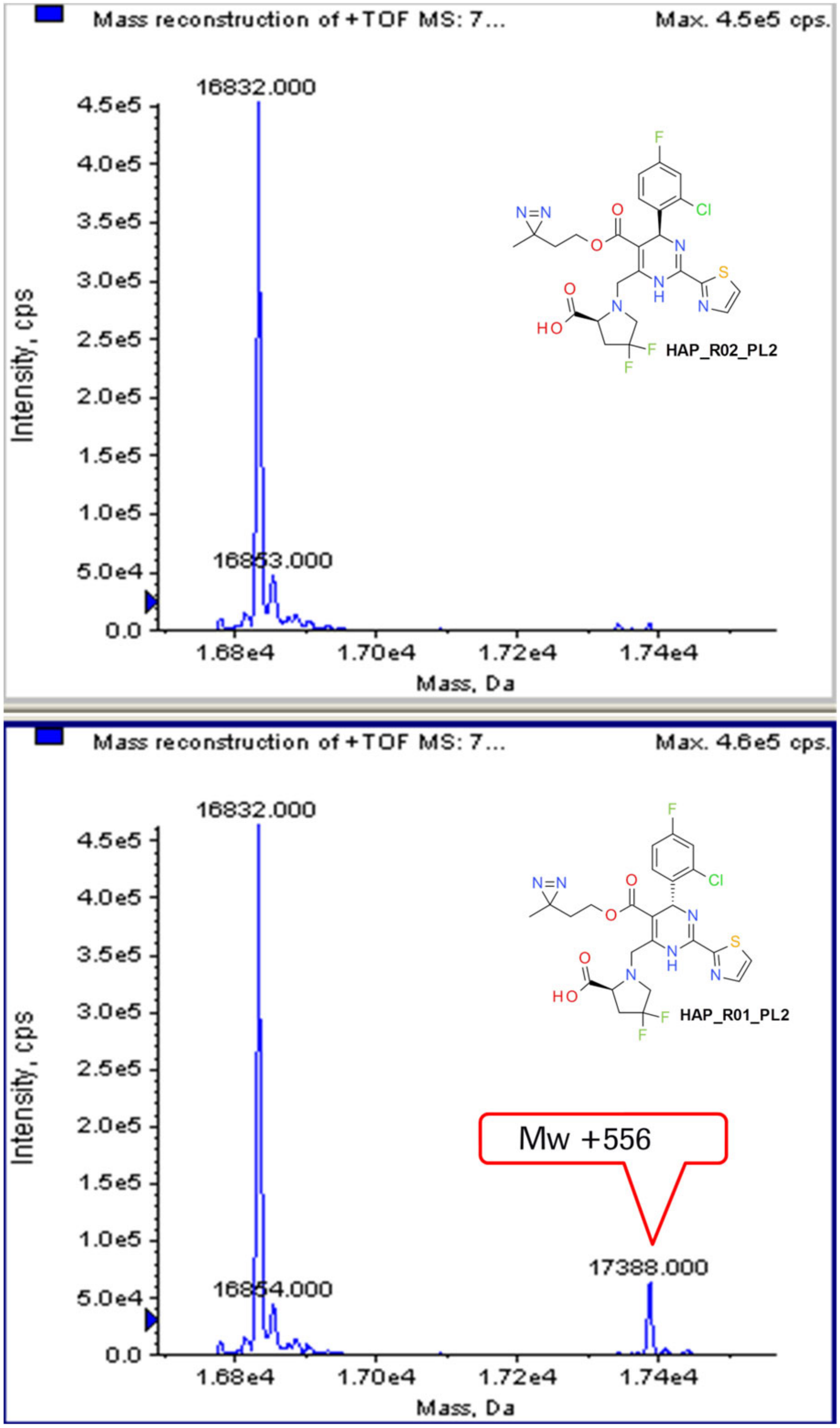

Supplementary Figure S4. Upper panel: Intact mass measurement of the inactive **HAP_R02_PL2**-treated sample. Bottom panel: Intact mass measurement of the active **HAP_R01_PL2**-treated sample showing covalent modification of core protein by the UV-activated label.

Supplementary Figure S5.

A Protease K-digested peptides

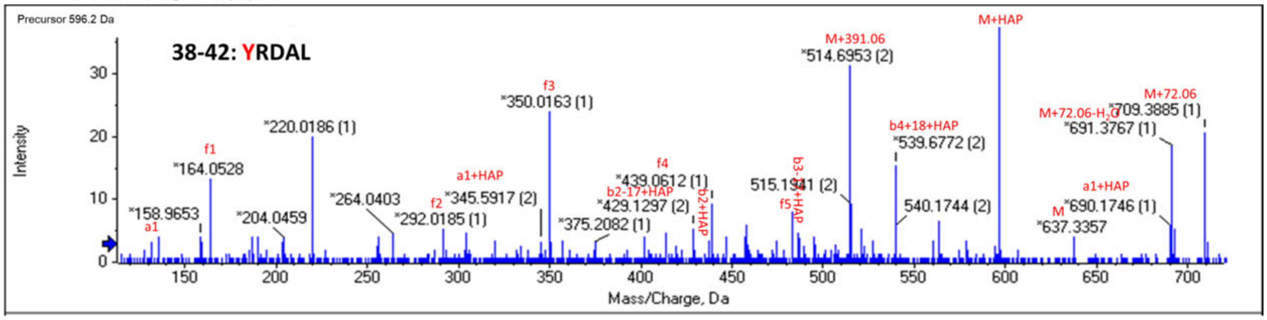

Sequence	Res. Mass	# (N)	A ion	A+PL2	A+2H +PL2	B ion	B+PL2	B+2H +PL2	B+2H +H2O+PL2	B+2H -NH3+PL2	Y ion	Y+PL2	# (C)
Y	163.06	1	136.08	690.18	345.59	164.07	718.18	359.59	368.60	351.08	637.33	1191.44	5
R	156.10	2	292.18	846.28	423.64	320.17	874.28	437.64	446.65	429.13	474.27	1028.37	4
D	115.03	3	407.20	961.31	481.16	435.20	989.31	495.15	504.16	486.64	318.17	872.27	3
A	71.04	4	478.24	1032.35	516.68	506.24	1060.34	530.67	539.68	522.16	203.14	757.25	2
L	113.08	5	591.33	1145.43	573.22	619.32	1173.43	587.21	596.22	578.70	132.10	686.21	1

B Trypsin-digested peptides

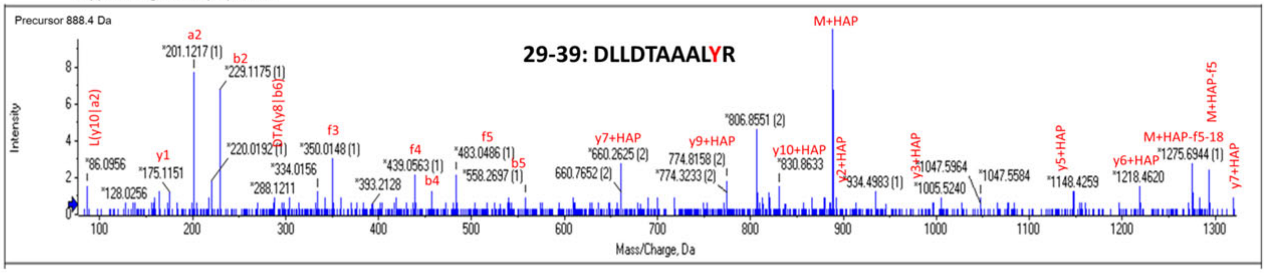

Symbol	Res. Mass	# (N)	A ion	B ion	Y ion	Y+PL2	Y+2H+PL2	# (C)
D	115.03	1	88.04	116.03	1221.65	1775.75	888.38	11
L	113.08	2	201.12	229.12	1106.62	1660.72	830.86	10
L	113.08	3	314.21	342.20	993.54	1547.64	774.32	9
D	115.03	4	429.23	457.23	880.45	1434.55	717.78	8
T	101.05	5	530.28	558.28	765.43	1319.53	660.27	7
A	71.04	6	601.32	629.31	664.38	1218.48	609.74	6
A	71.04	7	672.36	700.35	593.34	1147.44	574.22	5
A	71.04	8	743.39	771.39	522.30	1076.40	538.71	4
L	113.08	9	856.48	884.47	451.27	1005.37	503.19	3
Y	163.06	10	1019.54	1047.54	338.18	892.28	446.65	2
R	156.10	11	1175.64	1203.64	175.11	729.22	365.11	1

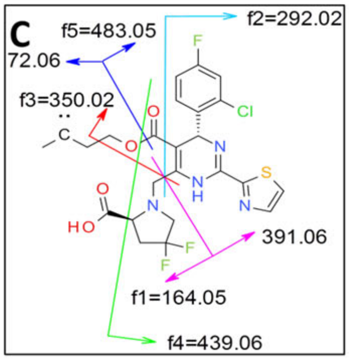

Supplementary Figure S5. Identification of photoaffinity labeling site Y38 by **HAP_R01_PL2**. (A) MS2 data of protease K-digested peptide 38-42: YRDAL. f1~f5 denotes observed fragment ions from **HAP_R01_PL2**. (B) MS2 data of trypsin-digested peptide 29-39: DLLDTAAALYR. (C) Fragmentation pattern of **HAP_R01_PL2**. f1~f5 indicate calculated fragment ions from UV-activated **HAP_R01_PL2.** Tables show the calculated mass of fragmented ions. Light blue denotes observed A or B ions. Blue color indicates the presence of photolabeled A or B ions. Light yellow denotes observed Y ions. Yellow color indicates the presence of photolabeled Y ions.

Supplementary Figure S6.

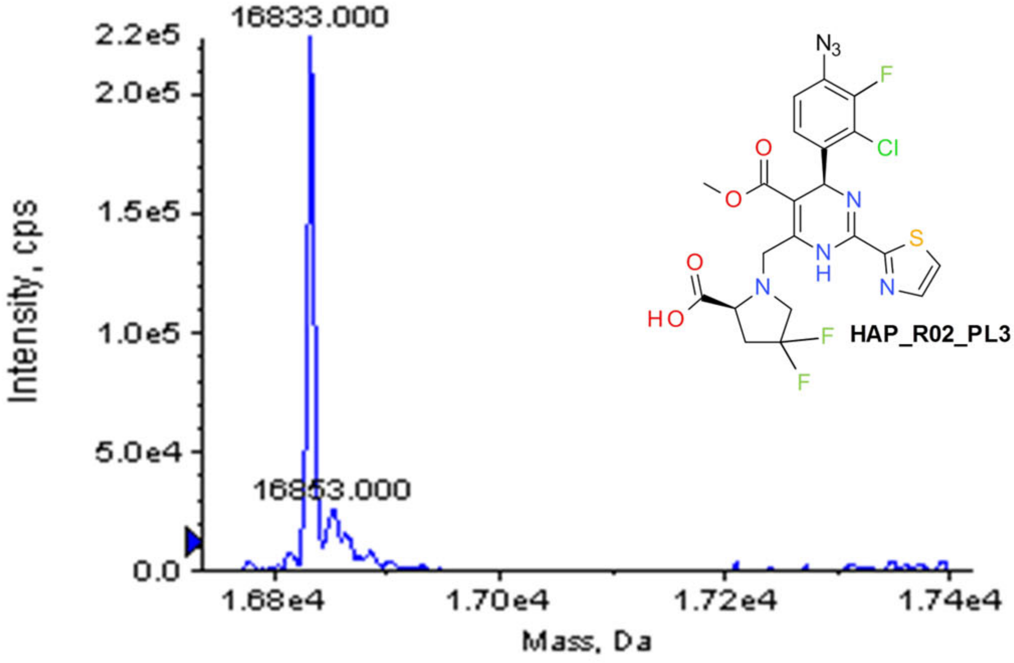

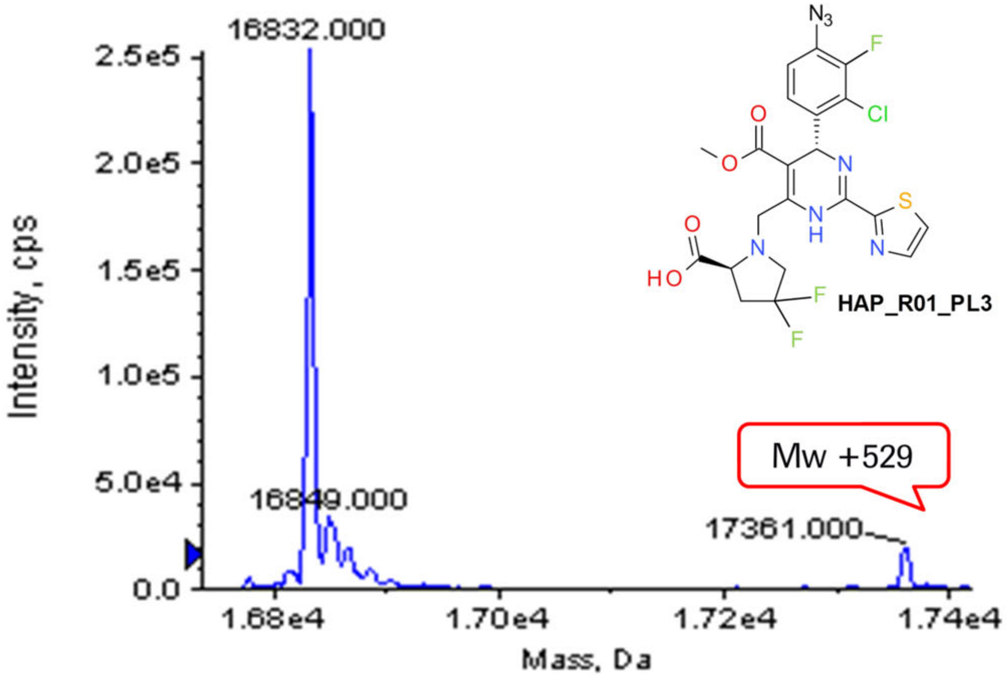

Supplementary Figure S6. Upper panel: intact mass measurement of the inactive **HAP_R02_PL3-**treated sample. Bottom panel: intact mass measurement of the active **HAP_R01_PL3**-treated sample showing covalent modification of core protein by the UV-activated label.

Supplementary Figure S7.

A Chymotrypsin-digested peptides

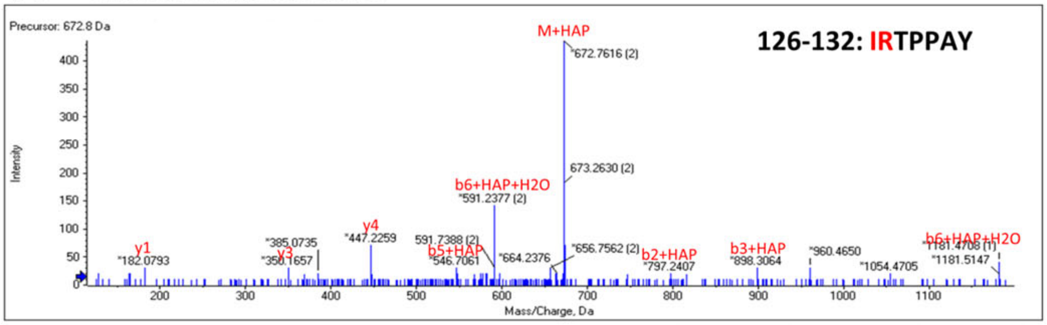

Symbol	Res. Mass	# (N)	A ion	B ion	B+PL3	B+PL3 +H2O	B+2H +PL3	B+2H +PL3+H2O	Y ion	Y+PL3	# (C)
I	113.08	1	30.03	114.09	641.16	659.17	321.08	330.09	817.46	1344.52	7
R	156.10	2	129.10	270.19	797.26	815.27	399.13	408.14	704.37	1231.43	6
T	101.05	3	315.18	371.24	898.30	916.32	449.66	458.66	548.27	1075.33	5
P	97.05	4	428.27	468.29	995.36	1013.37	498.18	507.19	447.22	974.28	4
P	97.05	5	584.37	565.35	1092.41	1110.42	546.71	555.71	350.17	877.23	3
A	71.04	6	685.41	636.38	1163.45	1181.46	582.23	591.23	253.12	780.18	2
Y	163.06	7	782.47	799.45	1326.51	1344.52	663.76	672.77	182.08	709.14	1

B Chymotrypsin-digested peptides

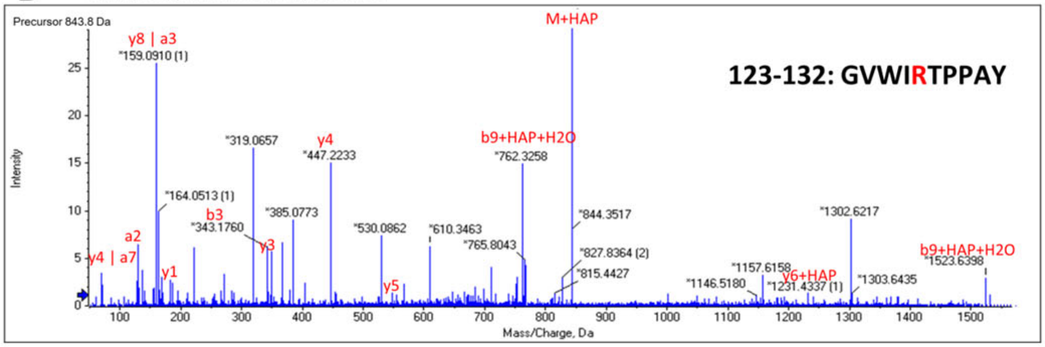

Symbol	Res. Mass	# (N)	A ion	B ion	B+PL3	B+PL3 +H2O	B+2H +PL3+H2O	Y ion	Y+PL3	# (C)
G	57.02	1	30.03	58.03	585.10	603.10	302.06	1159.63	1686.69	10
V	99.07	2	129.10	157.10	684.16	702.17	351.59	1102.60	1629.67	9
W	186.08	3	315.18	343.18	870.24	888.25	444.63	1003.54	1530.60	8
I	113.08	4	428.27	456.26	983.32	1001.34	501.17	817.46	1344.52	7
R	156.10	5	584.37	612.36	1139.43	1157.44	579.22	704.37	1231.43	6
T	101.05	6	685.41	713.41	1240.47	1258.49	629.75	548.27	1075.34	5
P	97.05	7	782.47	810.46	1337.53	1355.54	678.27	447.22	974.29	4
P	97.05	8	879.52	907.51	1434.58	1452.59	726.80	350.17	877.24	3
A	71.04	9	950.56	978.55	1505.62	1523.63	762.32	253.12	780.18	2
Y	163.06	10	1113.62	1141.62	1668.68	1686.69	843.85	182.08	709.15	1

Supplementary Figure S7. Identification of photoaffinity labeling site R127 by **HAP_R01_PL3**. The color scheme is the same as Fig. S6. (A) MS2 data of chymotrypsin-digested peptide 126-132: IRTPPAY. (B) MS2 data of chymotrypsin-digested peptide 123-132: GVWIRTPPAY.

Supplementary Figure S8.

Pepsin-digested peptides

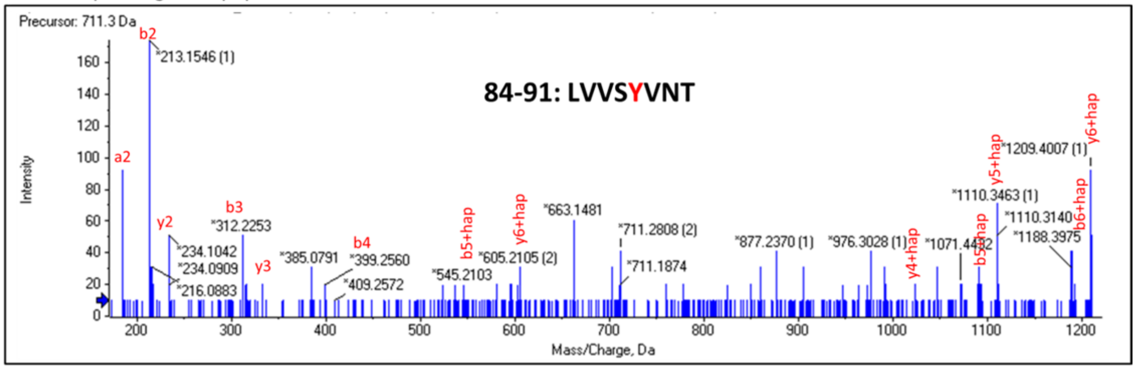

Symbol	Res. Mass	# (N)	A ion	B ion	B+PL3	B+PL3 +2H	Y ion	Y+PL3	Y+PL3 +2H	# (C)
L	113.08	1	86.10	114.09	641.16	321.08	894.49	1421.56	711.2829	8
V	99.07	2	185.17	213.16	740.22	370.62	781.41	1308.47	654.7408	7
V	99.07	3	284.23	312.23	839.29	420.15	682.34	1209.41	605.2066	6
S	87.03	4	371.27	399.26	926.33	463.67	583.27	1110.34	555.6724	5
Y	163.06	5	534.33	562.32	1089.39	545.20	496.24	1023.31	512.1564	4
V	99.07	6	633.40	661.39	1188.46	594.73	333.18	860.24	430.6247	3
N	114.04	7	747.44	775.44	1302.50	651.75	234.11	761.17	381.0905	2
T	101.05	8	848.49	876.48	1403.55	702.28	120.07	647.13	324.0691	1

B Pepsin-digested peptides

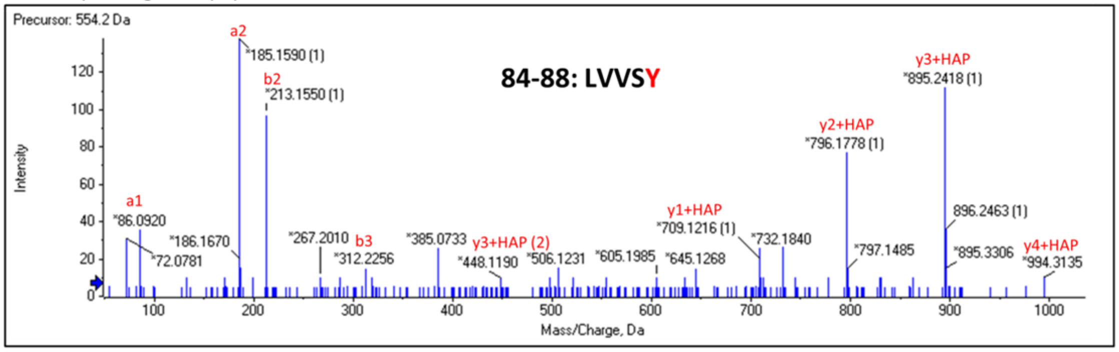

Symbol	Res. Mass	# (N)	A ion	B ion	B+PL3	Y ion	Y+PL3	Y+PL3 +2H	# (C)
L	113.08	1	86.10	114.09	641.16	580.33	1107.40	554.20	5
V	99.07	2	185.17	213.16	740.22	467.25	994.31	497.66	4
V	99.07	3	284.23	312.23	839.29	368.18	895.25	448.13	3
S	87.03	4	371.27	399.26	926.33	269.11	796.18	398.59	2
Y	163.06	5	534.33	562.32	1089.39	182.08	709.15	355.08	1

Supplementary Figure S8. Identification of photoaffinity labeling site Y88 by **HAP_R01_PL3** (A) and **HAP_R02_PL3** (B). The color scheme is the same as Fig. S6. (A) MS2 data of pepsin-digested peptide 84-91: LVVSYVNT. (B) MS2 data of pepsin-digested peptide 84-88: LVVSY.

Supplementary Figure S9.

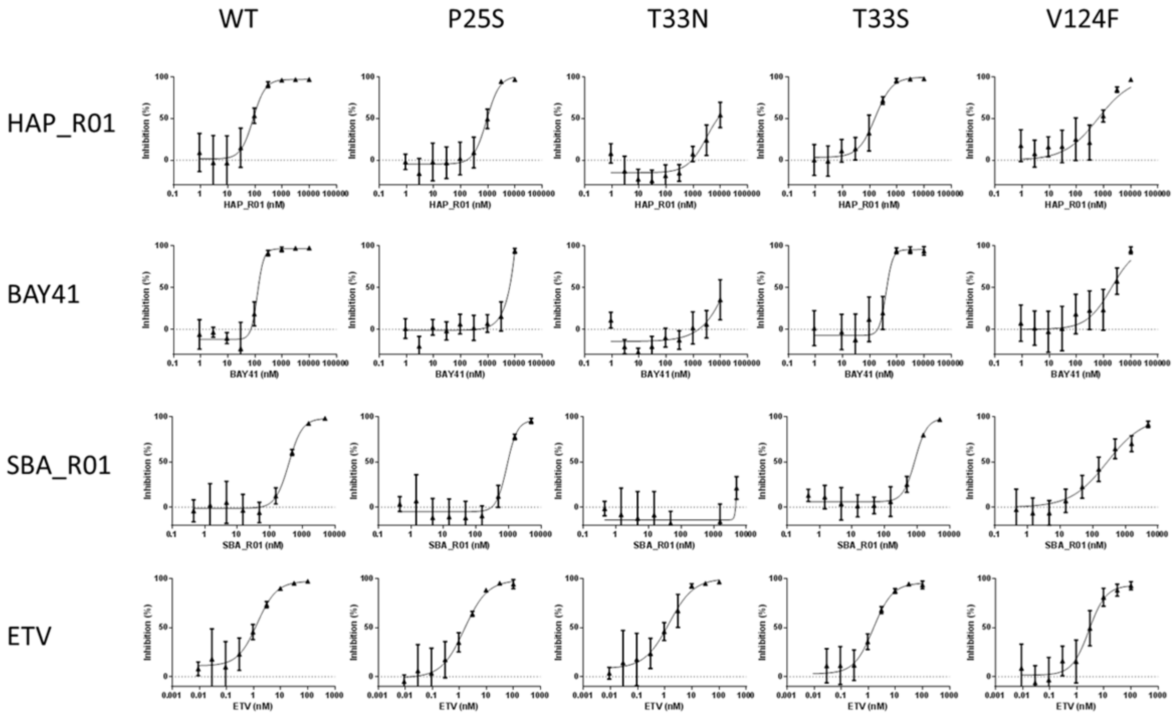

Supplementary Figure S9. Antiviral activity of HAP or SBA against HBV core mutants determined in the HepG2 HBV transient transfection assay. Mean dose response curves from three independent experiments were plotted for selected mutants. Percent inhibition values for drug treated samples were normalized to values from HepG2 cells only treated with DMSO.

Supplementary Figure S10.

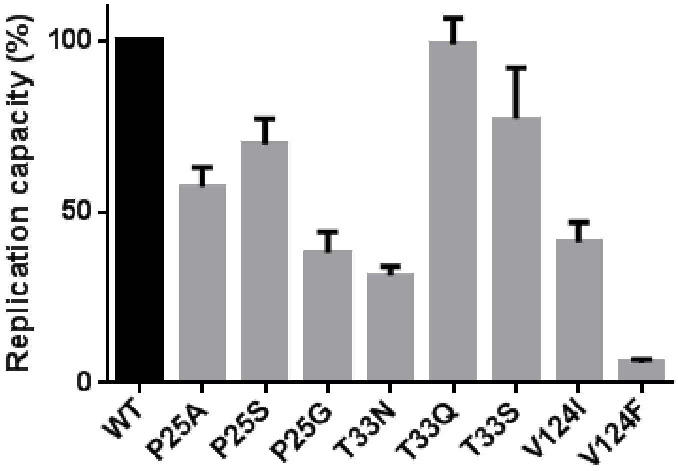

HBV core mutant	Mean HBV replication capacity ± SD (%)
WT	100.00
P25A	57.20 ± 5.86
P25S	69.74 ± 7.39
P25G	38.01 ± 6.16
T33N	31.55 ± 2.44
T33Q	98.98 ± 7.64
T33S	77.16 ± 14.8
V124I	41.16 ± 5.79
V124F	5.82 ± 1.13

Supplementary Figure S10. Replication capacity of HBV core mutants compared to wild type was determined in the HepG2 HBV transient transfection assay based on DNA copies. Shown are mean values ± standard deviation (SD) from three independent experiments.

Supplementary Figure S11.

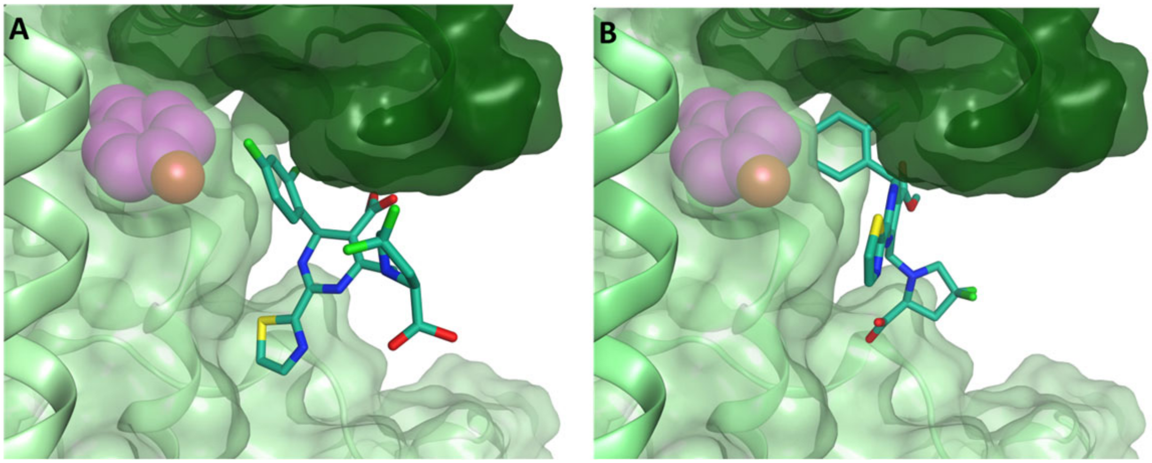

Supplementary Figure S11. Docking of **HAP_R01** and its corresponding *S*-diastereomer **HAP_R02** into the novel type 2 contact-to-spike interface. Light green represents the dimer in asymmetric unit. The symmetry mate is colored in dark green. Y88 is shown in space-filling model colored in magenta. Compounds are colored in cyan. Only the favorite docking poses are shown.

Supplementary Figure S12.

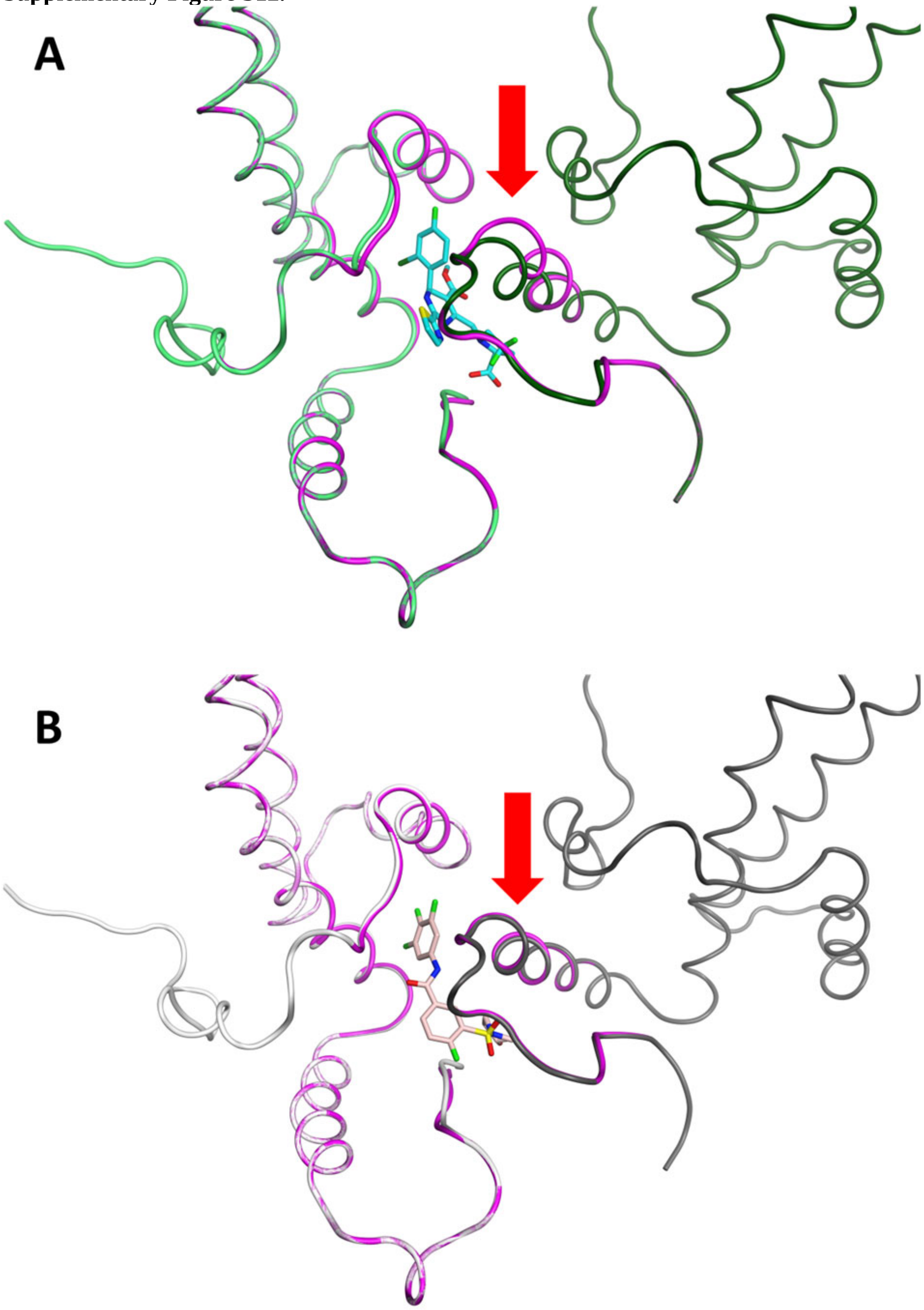

Supplementary Figure S12. Local conformational changes caused by **HAP_R01** during energy minimization. Chain C and chain D' of icosahedral T4 capsid was extracted from 1QGT. The ligands and interface residues within 8 Å radius were regarded as flexible during energy minimization. (A) Light green and dark green ribbons represent chain C and chain D' from 1QGT, respectively. After forcefield minimization, **HAP_R01** is highlighted in cyan stick and HBV core protein is shown in magenta. (B) Light grey and dark grey represent chain C and chain D' from 1QGT, respectively. After forcefield minimization, **SBA_R01** is coloured in pink and HBV core protein is also shown in magenta. The red arrows indicate that only **HAP_R01** can induce significant conformational changes on helix 5 of chain D' (S121-P129).

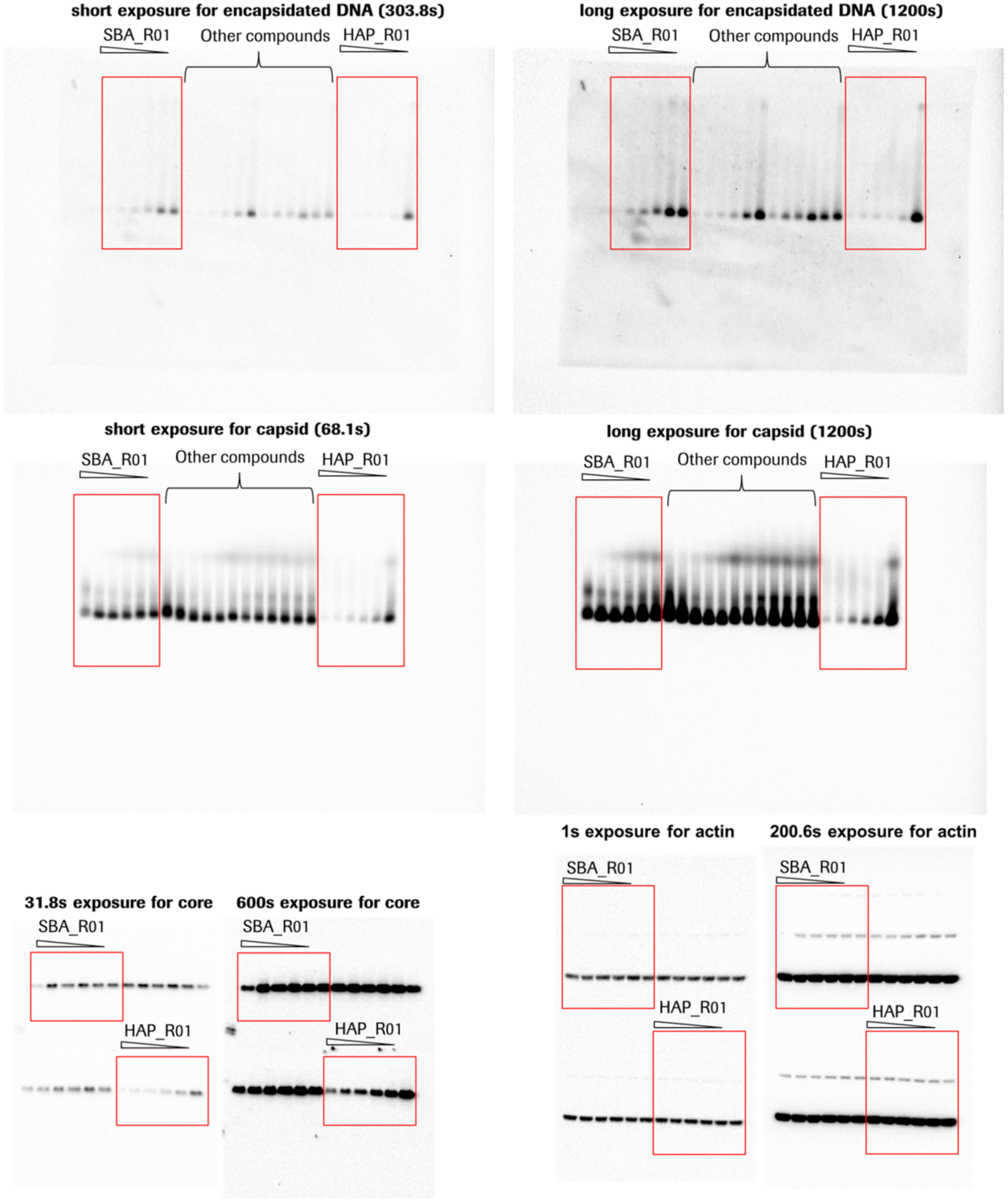

Supplementary Figure S13. The full-length blots for the DNA and native gels. The full length DNA gel (top row) and native capsid gel (middle row) were transfered to the whole membrane. Multiple images were taken under different exposures. The denatured gel (bottom row) electrophoresis was carried out to detect core protein and actin. After proteins were electrotransferred to a nitrocellulose membrane, the membrane was cut around the 30 KDa protein marker band. The upper parts of the membrane were used for β-actin (42 KDa) blotting and the lower parts were used for HBV core protein (~21 KDa) detection. After blotting, images were visualized by ChemiDoc™ imaging System under different exposures. Red boxes indicate the tested compounds **HAP_R01** and **SBA_R01** in this paper. Other unrelated compounds are not disclosed in this work.

Supplementary Tables

Supplementary Table S1. Data collection and refinement statistics.

	Y132A hexamer-HAP_R01	Y132A hexamer-SBA_R01	Apo Y132A
**Wavelength (Å)**	0.9792	0.9793	0.9791
**Resolution range (Å)**	32.36-1.95 (1.98-1.95)	39.04-1.69 (1.73-1.69)	32.51-2.62 (2.72-2.62)
**Space group**	P 1	P 1	P 41 21 2
**Unit cell**	65.24 66.93 87.07<br>68.28 69.02 83.62	63.93 68.05 85.70<br>68.49 69.99 83.49	79.27 79.27 163.14<br>90.00 90.00 90.00
**Unique reflections**	90046 (8027)	134045 (12255)	16156 (1538)
**Multiplicity**	4.1 (3.8)	4.0 (3.4)	10.3 (4.8)
**Completeness (%)**	97.1 (95.8)	95.9 (90.7)	99.0 (97.0)
**Mean I/sigma(I)**	8.0 (2.5)	10.5 (1.9)	15.25 (2.82)
**Wilson B-factor ($Å^2$)**	28.1	26.5	79.1
**R-merge (%)**	0.100 (0.762)	0.087 (1.219)	0.118 (0.734)
**R-meas (%)**	0.114 (0.883)	0.100 (1.431)	0.124 (0.818)
**R-pim (%)**	0.056 (0.443)	0.049 (0.738)	0.037 (0.390)
**CC1/2**	(0.768)	(0.498)	(0.61)
**R-work (%)**	0.207 (0.323)	0.180 (0.335)	0.207 (0.358)
**R-free (%)**	0.239 (0.370)	0.208 (0.325)	0.258 (0.440)
**No. of non-hydrogen atoms**	7742	8151	2275
**macromolecules**	6967	7298	2261
**ligands**	204	174	3
**water**	504	608	11
**Protein residues**	868	898	284
**RMS(bonds) (Å)**	0.010	0.010	0.009
**RMS(angles) (º)**	0.95	0.95	1.06
**Ramachandran favored (%)**	98.0	98.0	93.0
**Ramachandran outliers (%)**	0	0.11	0.36
**Clashscore**	5.08	3.65	13.38
**Average B-factor ($Å^2$)**	41.6	40.1	99.3
**Macromolecules ($Å^2$)**	41.2	39.6	99.4
**Ligands ($Å^2$)**	31.0	28.8	
**Solvent ($Å^2$)**	50.7	47.2	75.1

Statistics for the highest-resolution shell are shown in parentheses.

Supplementary Table S2.

Compound	IC50 (μM)	HepG2.2.15 EC50 (μM)
**HAP_R01_PL1**	1.15 ± 0.59	0.093 ± 0.049
**HAP_R02_PL1**	> 100	> 10
**HAP_R01_PL2**	1.32 ± 0.44	0.44 ± 0.06
**HAP_R02_PL2**	25.6 ± 11.1	> 10
**HAP_R01_PL3**	1.88 ± 0.66	0.23 ± 0.10
**HAP_R02_PL3**	> 100	> 10

Table S2. Activity of HAP and SBA photoaffinity labels. IC50 values of biochemical quenching assay and EC50 values of HepG2.2.15 antiviral assay are listed

Supplementary Methods

Protein expression and purification

The recombinant protein was expressed in Escherichia coli strain BL21(DE3). IPTG was added to a final concentration of 0.1mM when OD600 reached 0.9. The culture was grown at 16 ˚C overnight. Cells were harvested by centrifugation at 8000 g for 10 min. About 14 g cells were lysed by ultra-sonication in190 ml lysis buffer (50 mM Tris pH 9.0, 2 M urea, 250 mM NaCl, 1 mM DTT). After centrifugation at 15000 g for 30 min, the supernatant was applied to Ni affinity column (Chelating Sepharose Fast Flow, 20mL, GE healthcare). Tagged protein was eluted with lysis buffer containing 20-50 mM imidazole. The pooled fractions were dialyzed against 25 mM Tris pH 9.0, 2 mM DTT. The protein was further purified by anion-exchange chromatography (HiTrap Q HP, 20 ml, GE healthcare) with linear gradient of 0 to 1000 mM NaCl. The hexahistidine tag was removed using tobacco etch virus (TEV) protease digestion over night at 30 ℃ (500 U/mg). The cleaved sample went through a second affinity column and flow-through containing tagless protein (with additional C-terminal ENLYFQ) was collected. The protein was dialyzed into 25 mM Tris buffer pH 9.0 containing 2 mM DTT and 10 mM EDTA. Finally the product was concentrated to 17 mg/ml and stored at -80˚C.

Capsid assembly fluorescence quenching assay

20 fold molar excess of maleimidyl BoDIPY-FL dye (Sigma) was added to Cp150 dimer overnight at 4°C. DMSO and unreacted dye were removed by using G25 column in ice-cold 50 mM HEPES pH7.5 buffer. The modified protein is referred to as C150Bo. The concentration of bound dye and labeled protein were determined by formula as follow:

[BoDIPY-FL]=A504/78,000 M^{-1}

[C150Bo] = (A280-[BoDIPY-FL]×1,300M^{-1})/60,900 M^{-1}

Pseudo-critical concentration of free dimer for assay was determined by adding NaCl at final concentration of 150 mM into various concentrations of C150Bo in 50 mM HEPES buffer pH 7.5. After 1 hour incubation at room temperature, the fluorescence values were read at λex = 485 nm and λem = 535 nm. The full assembly control of capsid was determined by mix 5 µM C150Bo with 1 M NaCl in 50mM HEPES, and for no-assembly controls, the fluorescence signal of 5 µM C150Bo only in 50 mM HEPES was measured. The concentration of the C150Bo dimer used in assay was selected when achieving 25−30% capsid assembly.

To evaluate effect of inhibitors on capsid assembly, assay was performed in 2% w/v Non-fat dried milk treated black, flat-bottomed 96-well black plates. Multiple doses of inhibitors were added in 5 µM C150Bo in 50 mM

HEPES buffer pH 7.5, and incubated at room temperature for 30 minutes. Then NaCl (at final concentration of 150 mM) in 50 mM HEPES buffer pH 7.5 was added to each sample well. After 1 hour incubation at RT, the fluorescence values were read at λex = 485 nm and λem = 535 nm with FlexStation. The fluorescence value was converted to assembly percentage with formula below.

$$\%\ assembly = 100 \times \frac{F_{25\%\ assembly} - F_{sample}}{F_{25\%assembly} - F_{full\text{-}assembly}}$$

Data were analysed by GraphPad Prism 6 using log(inhibitor) vs. normalized response -- Variable slope.

HepG2.2.15 anti-HBV assay

HepG2.2.15 cells carrying the HBV genome were seeded into 96-well plates at a density of 3×10^4 cells per well in 100 µL DMEM/F12 media(Life Science) supplemented with 2.5% fetal bovine serum, 20 mM L-glutamine, 0.1 mM NEAA, 50 U/mL penicillin, 50 U/mL streptomycin and cultured overnight at 37 ºC. The test compounds were serially half-log diluted in DMSO, then diluted 100 times in culture media. 100 µL diluted solution of tested compounds were added into the plates to reach 0.5% final concentration of DMSO in every well. Five days after compound treatment, culture supernatant was collected for further analysis.

For quantitative PCR detection of extracellular HBV DNA, culture supernatant was processed by 500 µg/ml Proteinase K (Sigma) digestion at 50 ˚C for 1 hour. After heat inactivation of the enzyme at 95 ˚C for 15 minutes, the samples were subjected to HBV DNA quantification by qPCR. The effective compound concentration at 50% HBV replication inhibition (EC50) was determined.

Cytotoxicity assay

Confluent HepG2.2.15 cells were trypsinized and split into 96-well plate at 5,000 cells/well with 100 µL/well and incubated in 37 ˚C, 5% CO_2 overnight. The next day, half-log series dilutions of testing compounds were prepared using medium and added 100 µL compounds into appropriate wells. For 3 days incubation, the media was replaced with fresh media (with compounds), then continued to incubate for two days. The cytotoxicity was measured using CCK-8 kit (Dongjindo)

Labeling of capsid with photoreactive HAP compounds

5 µM core protein dimer and 20 µM HAP compound were mixed in a buffer containing 50 mM HEPES, 250 mM NaCl, pH 7.4 to prepare sample with the dimer : probe ratio of 1:4. 1:2 and 1:10 ratios were also tested for the **PL1**s to determine the optimal labeling condition. Due to similar labeling efficiency with three tested concentrations, the dimer : probe molar ratio of 1:4 was adopted for **PL2**s and **PL3**s. The solution was then irradiated for 30 minutes with light from a mercury lamp (medium pressure, 500 W). The carbene group

generated by UV light exposure is supposed to covalently modify amino acid residue(s) in its close vicinity.

Protease digestion

The irradiated sample was digested with four proteases, i.e. trypsin, chymotrypsin, proteinase K and pepsin, respectively. Briefly, about 20 μL of sample was mixed with 100 ul of 8 M urea in 50 mM ammonium bicarbonate buffer (ABC) in an Amicon® Ultra-0.5 centrifugal filter (10K Mw cutoff, Millipore). The preparation was centrifuged at 14000 g for 15 min. Then 200 μL of 50 mM ABC (0.06 M HCl was added for pepsin digestion) was added and centrifuged again. Proteases were added according to vendor's instruction. The preparation was incubated at 37°C overnight. The resultant peptide mixture was collected to a new tube by centrifugation with 100 μL 50% acetonitrile in 50 mM ABC and 100 μL of 50% acetonitrile in 50% formic acid, respectively. The reaction mixture was dried in a speed vac and reconstituted with 30 μL of 5% DMSO/5% formic acid/5% acetonitrile.

LC/MS

3 ul of irradiated sample was injected onto a reverse phase C4 column (BEH300, 150×2.1 mm, 1.7 μm, Waters) equilibrated at 75°C at a flow rate of 250 μL/min. Reverse phase chromatography was performed using an ultra-performance liquid chromatography (UPLC) system (Acquity UPLC, Waters). The gradient was generated by using 0.1% formic acid for mobile phase A and acetonitrile containing 0.1% FA for mobile phase B. After an isocratic elution at 20% B for 2 min, B was raised to 60% in 5 min and to 85% in an additional 1 min. The column was then washed for 2 min at 85% B and re-equilibrated for 5 min at 20% B, giving an overall run time of 15 min. The eluted species were then analyzed online by a Q-TOF mass spectrometer (TripleTOF™ 5600, AB Sciex) operating in the positive ion mode from m/z 400–3000 and calibrated according to the manufacturer's procedure.

LC/MS/MS

An aliquot of the digestion representing an amount of 1 μg core protein was injected onto a reverse phase C18 column (BEH300, 150×2.1 mm, 1.7 um, Waters) equilibrated at 35°C at a flow rate of 160 μL/min. The gradient was generated by using 0.1% formic acid for mobile phase A and acetonitrile containing 0.1% FA for mobile phase B. After an isocratic elution at 5% B for 2 min, B was raised to 45% in 29 min and to 90% in an additional 5 min. The column was then washed for 4 min at 90% B and re-equilibrated for 10 min at 5% B, giving an overall run time of 50 min. LC/MS/MS was performed in an information dependent mode (IDA) using the same system as above. For each cycle, one full MS scan was followed by up to 10 MS/MS for the most intense ions.

Energy minimization.

In capsid crystal structures in complex with **HAP1** and **AT-130**, the C-D' interface is the shared ligand binding site (Fig. 5C) [1,2]. Since the compound binding pockets in Y132A hexamer structures are dominated by the concave from chain B, we simply superimposed the chain B of the Y132A-compound structures onto the chain C of T4 capsid 1QGT to generate the initial positions of the ligand. During energy minimization, ligands and residues within 8 Å or 20 Å radius were kept free of restraints. The local minimum of the molecular energy function was calculated using Amber12EHT forcefield till RMS gradient fell below 0.1 kcal/mol/Å^2.

Rational design and synthesis of photoreactive HAPs

Diazirine has been widely used in photolabeling studies due to its small size and good stability [3]. **HAP_R01** has a difluoroproline moiety at its 6-position, and the gem-difluoro group is critical to its high activities in both biochemical and cellular assays. Given the fact that diazirine and difluoromethyl have similar size and lipophilicity, we considered these two groups as bioisosteres, and prepared **HAP_R01_PL1** where the original difluoromethyle was replaced by photoreactive group diazirine. To our delight, compound 1 did exhibit good activity in the biochemical assay and excellent anti-HBV activity in the cellular assay (Tab. S2). Since short alkyl esters were found tolerated at the 5th position of HAP, a butyl chain bearing a diazirine group was incorporated into that position to give **HAP_R01_PL2**. Phenyl ring at the 4th position of HAP is indispensable for its capsid targeting activity, and the small sized and linear substituent, cyano group, at the para-position of the phenyl ring was well tolerated in terms of potency. Due to the similarity of azido and cyano groups, azido group was envisioned as the best choice for photoreactive group at this direction. In addition, fluorine at the meta-position was beneficial to capsid assembly activity. Thus the third probe, **HAP_R01_PL3**, was prepared with a 4-azido-3-fluoro-2-chlorophenyl fragment at the 4th position of the molecule. The synthesis of all these probes was based on a three component reaction between an acetoacetate derived ester, a substituted benzaldehyde and thiazole-2-carboxamidine hydrochloride .

Synthesis of HAP_R01_PL1 and HAP_R02_PL1

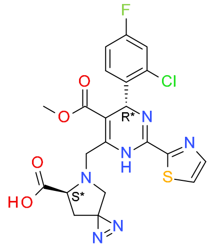

HAP_R01_PL1

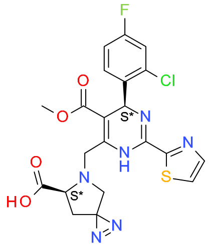

HAP_R02_PL1

Synthetic scheme 1

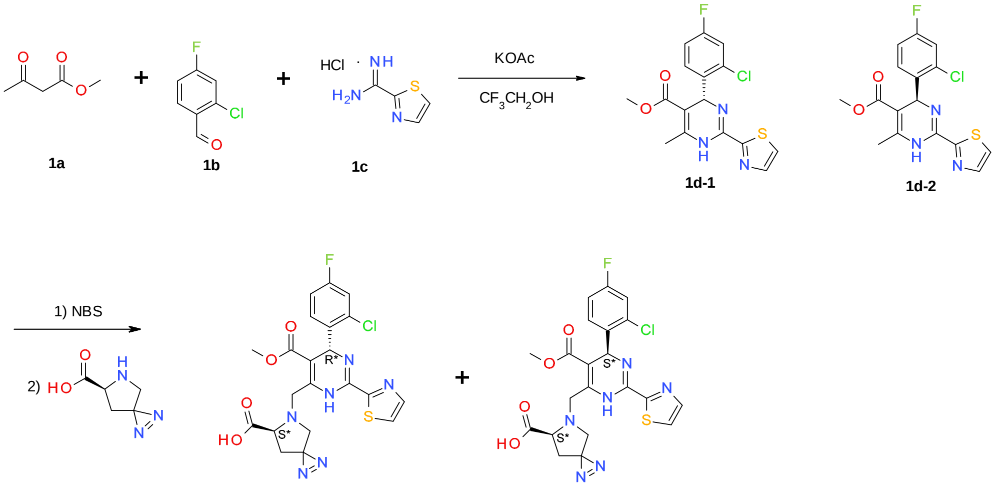

Preparation of methyl (4R)-4-(2-chloro-4-fluoro-phenyl)-6-methyl-2-thiazol-2-yl-1,4-dihydropyrimidine-5-carboxylate (compound 1d-1) and methyl (4S)-4-(2-chloro-4-fluoro-phenyl)-6-methyl-2-thiazol-2-yl-1,4-dihydropyrimidine-5-carboxylate (compound 1d-2)

To a stirred solution of methyl acetoacetate (compound **1a**, 0.12 g, 1.0 mmol), 2-chloro-5-fluorobenzaldehyde (compound **1b**, 0.16 g, 1.0 mmol) and thiazole-2-carboxamidine hydrochloride (compound **1c**, 0.13 g, 1.0 mmol) in CF_3CH_2OH (8.0 mL) was added potassium acetate (0.20 g, 2.0 mmol). The reaction mixture was refluxed for 16 hours. After cooled to room temperature, the reaction mixture was concentrated and the residue was dissolved in ethyl acetate and washed with brine. The organic layer was dried over Na_2SO_4 and concentrated, and the residue was purified by column chromatography to afford 4-(2-chloro-4-fluoro-phenyl)-6-methyl-2-thiazol-2-yl-1,4-dihydro-pyrimidine-5-carboxylic acid methyl ester as a yellow solid (310 mg), which was subjected to supercritical fluid chromatography (SFC) chiral separation to give methyl (4R)-4-(2-chloro-4-fluoro-phenyl)-6-methyl-2-thiazol-2-yl-1,4-dihydropyrimidine-5-carboxylate (compound **1d-1**, 0.12 g) {$[\alpha]D_{20}$ -55.0 (c 0.845, MeOH)} and methyl (4S)-4-(2-chloro-4-fluoro-phenyl)-6-methyl-2-thiazol-2-yl-1,4-dihydropyrimidine-5-carboxylate (compound **1d-2**, 0.13 g) {$[\alpha]D_{20}$ +44.6 (c 0.175, MeOH)}. LCMS ($M+H^+$): 366. ^1H NMR (DMSO-d6, 400 MHz): δ ppm 9.98 (s, 1H), 7.97 (d, J = 4.0 Hz, 1H), 7.90 (d, J = 4.0 Hz, 1H),

7.41 (dd, J = 8.0, 4.0 Hz, 1H), 7.35 (dd, J = 8.0, 8.0 Hz, 1H), 7.18 (td, J = 8.0, 4.0 Hz, 1H), 5.98 (s, 1H), 3.53 (s, 3H), 2.47 (s, 3H).

Preparation of (5S)-6-[[(4R)-4-(2-chloro-4-fluoro-phenyl)-5-methoxycarbonyl-2-thiazol-2-yl-1,4-dihydropyrimidin-6-yl]methyl]-1,2,6-triazaspiro[2.4]hept-1-ene-5-carboxylic acid (HAP_R01_PL1)

To a stirred solution of methyl (4R)-4-(2-chloro-4-fluoro-phenyl)-6-methyl-2-thiazol-2-yl-1,4-dihydropyrimidine-5-carboxylate (compound **1d-1**, 73 mg, 0.2 mmol) in CCl_4 (5 mL) was added N-bromosuccinimide (40 mg, 0.22 mmol) in portions. The reaction mixture was stirred at room temperature for 1 hour, and then washed with saturated $NaHCO_3$ solution and brine. The organic phase was seperated and dried over Na_2SO_4, filtrated and concentrated. The residue was dissolved in DMF (5 mL), to it were added 1,2,6-triazaspiro[2.4]hept-1-ene-5-carboxylic acid (56 mg, 0.4 mmol) and DIPEA (78 mg, 0.6 mmol). The resulting reaction mixture was stirred at room temperature for 2 hours. Then the reaction mixture was purified by prep-HPLC to give **HAP_R01_PL1** as a light yellow solid (53 mg). LCMS (M+H)+: 505. ^{1}H NMR (400MHz, METHANOL-d4) δ: 8.11 - 8.05 (m, 1H), 7.98 (d, J=3.0 Hz, 1H), 7.54 (dd, J=6.0, 8.7 Hz, 1H), 7.30 (dd, J=2.6, 8.7 Hz, 1H), 7.12 (dt, J=2.6, 8.4 Hz, 1H), 6.25 - 6.20 (m, 1H), 4.71 - 4.56 (m, 2H), 4.50 (dd, J=8.6, 16.1 Hz, 1H), 3.65 (s, 3H), 3.26 (d, J=12.3 Hz, 1H), 3.05 (d, J=12.3 Hz, 1H), 2.24 (dd, J=8.6, 14.7 Hz, 1H), 2.09 - 1.96 (m, 1H).

Preparation of (5S)-6-[[(4S)-4-(2-chloro-4-fluoro-phenyl)-5-methoxycarbonyl-2-thiazol-2-yl-1,4-dihydropyrimidin-6-yl]methyl]-1,2,6-triazaspiro[2.4]hept-1-ene-5-carboxylic acid (HAP_R02_PL1)

HAP_R02_PL1 was prepared from compound **1d-2** according to the procedure of **HAP_R01_PL1**. **HAP_R02_PL1** was obtained as a light yellow solid (56 mg). LCMS (M+H)+: 505. ^{1}H NMR (400MHz, METHANOL-d4) δ: 8.12 - 8.07 (m, 1H), 8.00 (d, J=3.1 Hz, 1H), 7.58 (dd, J=6.0, 8.7 Hz, 1H), 7.31 (dd, J=2.6, 8.7 Hz, 1H), 7.13 (dt, J=2.7, 8.4 Hz, 1H), 6.22 (s, 1H), 4.78 - 4.68 (m, 1H), 4.59 - 4.49 (m, 2H), 3.65 (s, 3H), 3.21 (d, J=12.3 Hz, 1H), 3.03 (d, J=12.3 Hz, 1H), 2.25 (dd, J=8.6, 14.7 Hz, 1H), 2.09 - 1.99 (m, 1H).

Synthesis of HAP_R01_PL2 and HAP_R02_PL2

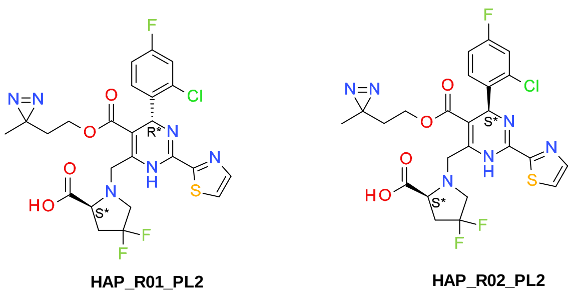

Synthetic scheme 2

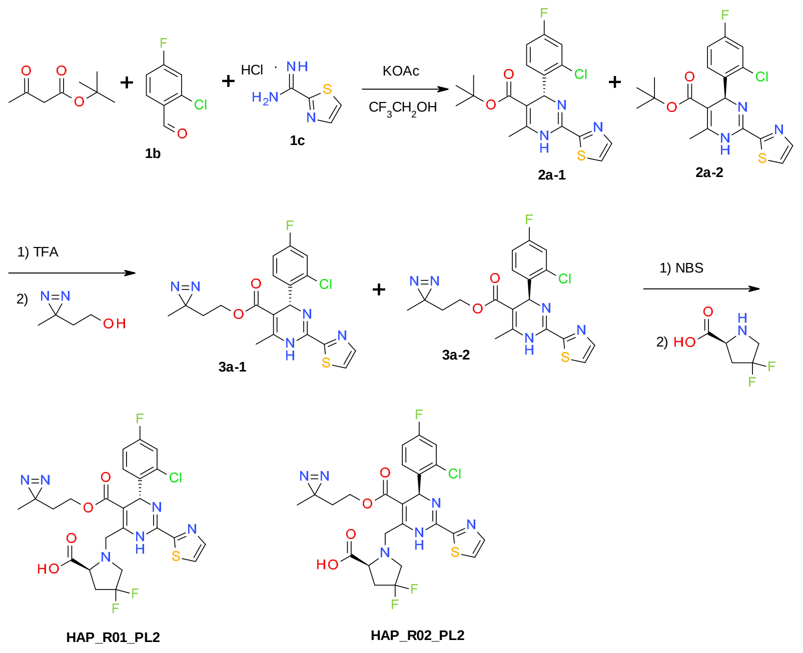

Preparation of Tert-butyl (4R)-4-(2-chloro-4-fluoro-phenyl)-6-methyl-2-thiazol-2-yl-1,4-dihydropyrimidine-5-carboxylate (compounds **2a-1** and **2a-2**)

compounds **2a-1** and **2a-2**were prepared in analogy to compounds **1d-1** and **1d-2** by using tert-butyl 3-oxobutanoate instead of methyl 3-oxobutanoate. LCMS (M+H)+: 408. ^{1}H NMR (400MHz, CHLOROFORM-d) δ: 7.83 (d, *J*=3.0 Hz, 1H), 7.49 (br s, 1H), 7.35 (dd, *J*=6.1, 8.7 Hz, 1H), 7.16 (dd, *J*=2.8, 8.5 Hz, 1H), 6.96 (dt, *J*=2.5, 8.3 Hz, 1H), 6.14 (s, 1H), 2.54 (s, 3H), 1.32 (s, 9H).

Preparation of 2-(3-methyldiazirin-3-yl)ethyl (4R)-4-(2-chloro-4-fluoro-phenyl)-6-methyl-2-thiazol-2-yl-1,4-dihydropyrimidine-5-carboxylate (compound 3a-1)

A mixture of tert-butyl (4R)-4-(2-chloro-4-fluoro-phenyl)-6-methyl-2-thiazol-2-yl-1,4-dihydropyrimidine-5-carboxylate (compound **2a-1**, 3.5 g, 8.6 mmol), trifluoroacetic acid (10 mL) and dichloromethane (5 mL) was stirred at room temperature for 3 hours. The reaction mixture was concentrated. The residue was partitioned between ethyl acetate and ice-water, and neutralized to pH ~4 with 1 N sodium hydroxide solution. The organic layer was separated, and the aqueous phase was extracted with ethyl acetate three times. The combined organic phase was dried over Na_2SO_4, filtrated and concentrated to give 2.4 g of a crude product (LCMS (M+H$^+$) 352). Thus obtained crude material (505 mg, 1.4 mmol) was dissolved in N,N-dimethylmethanamide (2 mL), to it were added 2-(3-methyldiazirin-3-yl)ethanol (300 mg, 3.0 mmol), 1-ethyl-3-[3-dimethylaminopropyl]carbodiimide hydrochloride (414 mg, 2.2 mmol) and 4-dimethylaminopyridine (527 mg, 4.3 mmol). The reaction mixture was stirred at 60 ^{o}C for 3 hours. The reaction mixture was cooled down and quenched by adding ice-water. The mixture was extracted with petroleum there/ethyl acetate (v/v, 2/1) three times. The combined organic phase was dried over Na_2SO_4, filtrated and concentrated to give crude compound **3a-1** (606 mg). LCMS (M+H$^+$): 434.

Preparation of (2S)-1-[[(4R)-4-(2-chloro-4-fluoro-phenyl)-5-[2-(3-methyldiazirin-3-yl)ethoxycarbonyl]-2-thiazol-2-yl-1,4-dihydropyrimidin-6-yl]methyl]-4,4-difluoro-pyrrolidine-2-carboxylic acid (HAP_R01_PL2)

The solution of 2-(3-methyldiazirin-3-yl)ethyl (4R)-4-(2-chloro-4-fluoro-phenyl)-6-methyl-2-thiazol-2-yl-1,4-dihydropyrimidine-5-carboxylate (compound **3a-1**, 606 mg, 1.4 mmol) in tetrachloromethane (10 mL) was stirred at 80 °C for 30 minutes, then N-bromosuccinimide (250 mg, 1.4 mmol) was added. The resulting mixture was stirred at 80 °C for another 30 minutes. The reaction mixture was cooled down, and washed with saturated $NaHCO_3$ solution and brine. The organic phase was seperated and dried over Na_2SO_4, filtrated and concentrated. The residue was disolved in dicloromethane (20 mL), followed by addition of (2S)-4,4-difluoropyrrolidine-2-carboxylic acid (371 mg, 1.4 mmol) and N,N-diisopropylethylamine (1.0 mL). The reaction mixture was stirred at 42 °C for 2 hours. The reaction mixture was cooled down and quenched by adding ice-water. The mixture was extracted with DCM three times. The combined organic phase was dried over Na_2SO_4, filtrated and concentrated. The residue was purified by prepare-HPLC to give **HAP_R01_PL2** (30 mg). LCMS (M+H+): 583. ^{1}H NMR (400MHz, METHANOL-d4) δ: 7.94 (d, *J*=3.3 Hz, 1H), 7.75 (d, *J*=3.0 Hz, 1H), 7.48 (dd, *J*=6.0, 8.8 Hz, 1H), 7.24 (dd, *J*=2.6, 8.7 Hz, 1H), 7.08 (dt, *J*=2.5, 8.4 Hz, 1H), 6.20 (s, 1H), 4.48 (d, *J*=16.6 Hz, 1H), 4.26 (d, *J*=16.6 Hz, 1H), 4.03 - 3.91 (m, 3H), 3.67 (q, *J*=11.3 Hz, 1H), 3.30 - 3.18 (m, 1H), 2.88 - 2.75 (m, 1H), 2.67 - 2.52 (m, 1H), 1.61 (t, *J*=6.1 Hz, 2H), 0.93 (s, 3H).

Preparation of (2S)-1-[[(4S)-4-(2-chloro-4-fluoro-phenyl)-5-[2-(3-methyldiazirin-3-yl)ethoxycarbonyl]-2-thiazol-2-yl-1,4-dihydropyrimidin-6-yl]methyl]-4,4-difluoro-pyrrolidine-2-carboxylic acid (HAP_R02_PL2)

HAP_R02_PL2 was prepared in analogy to **HAP_R01_PL2** from compound **2a-2** instead of **2a-1**. LCMS (M+H+): 583. ^{1}H NMR (400MHz, METHANOL-d4) δ: 8.12 (d, *J*=3.0 Hz, 1H), 8.05 (d, *J*=3.0 Hz, 1H), 7.60 (dd, *J*=6.0, 8.8 Hz, 1H), 7.32 (dd, *J*=2.5, 8.5 Hz, 1H), 7.14 (dt, *J*=2.5, 8.4 Hz, 1H), 6.31 (s, 1H), 4.65 (d, *J*=17.3 Hz, 1H), 4.25 (d, *J*=17.1 Hz, 1H), 4.10 (dd, *J*=7.9, 8.7 Hz, 1H), 4.02 (dt, *J*=2.1, 6.1 Hz, 2H), 3.72 - 3.62 (m, 1H), 3.31 - 3.21 (m, 1H), 2.95 - 2.81 (m, 1H), 2.66 - 2.52 (m, 1H), 1.64 (t, *J*=6.3 Hz, 2H), 0.95 (s, 3H).

Synthesis of HAP_R01_PL3 and HAP_R02_PL3

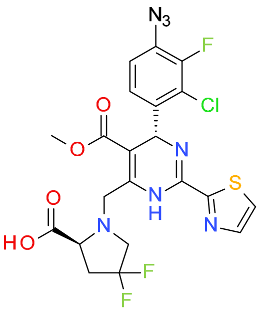

HAP_R01-PL3

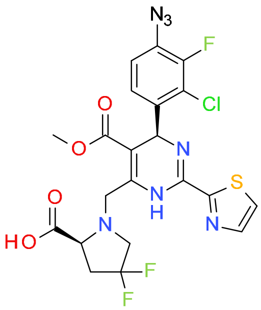

HAP_R02-PL3

HAP_R01_PL3 and **HAP_R02_PL3** were prepared in analogy to **HAP_R01_PL1** and **HAP_R02_PL1** by using 4-azido-2-chloro-3-fluoro-benzaldehyde instead of 2-chloro-4-fluoro-benzaldehyde.

HAP_R01_PL3 , a yellowish solid. LCMS (M+H$^+$): 556. ^{1}H NMR (400MHz, METHANOL-d4) δ: 7.93 (d, *J*=3.3 Hz, 1H), 7.75 (d, *J*=3.3 Hz, 1H), 7.27 (dd, *J*=1.5, 8.5 Hz, 1H), 7.16 (t, *J*=8.0 Hz, 1H), 6.14 (s, 1H), 4.42 (d, *J*=16.6 Hz, 1H), 4.19 (d, *J*=16.6 Hz, 1H), 3.95 (t, *J*=8.0 Hz, 1H), 3.70 - 3.57 (m, 4H), 3.27 - 3.14 (m, 1H), 2.87 - 2.73 (m, 1H), 2.65 - 2.50 (m, 1H).

HAP_R02_PL3, a yellowish solid. LCMS ($M+H^+$): 556. ^1H NMR (400MHz, METHANOL-d4) δ: 7.94 (d, *J*=3.3 Hz, 1H), 7.76 (d, *J*=3.3 Hz, 1H), 7.29 (dd, *J*=1.8, 8.5 Hz, 1H), 7.13 (t, *J*=8.3 Hz, 1H), 6.15 (s, 1H), 4.49 (d, *J*=16.8 Hz, 1H), 4.17 (d, *J*=16.8 Hz, 1H), 3.94 (t, *J*=8.2 Hz, 1H), 3.62 - 3.52 (m, 4H), 3.22 - 3.08 (m, 1H), 2.87 - 2.74 (m, 1H), 2.65 - 2.49 (m, 1H).

Supplementary References

1 Bourne, C. *et al.* Small-molecule effectors of hepatitis B virus capsid assembly give insight into virus life cycle. *J Virol* **82**, 10262-10270, doi:10.1128/JVI.01360-08 (2008).

2 Katen, S. P., Tan, Z., Chirapu, S. R., Finn, M. G. & Zlotnick, A. Assembly-directed antivirals differentially bind quasiequivalent pockets to modify hepatitis B virus capsid tertiary and quaternary structure. *Structure* **21**, 1406-1416, doi:10.1016/j.str.2013.06.013 (2013).

3 Dubinsky, L., Krom, B. P. & Meijler, M. M. Diazirine based photoaffinity labeling. *Bioorg Med Chem* **20**, 554-570, doi:10.1016/j.bmc.2011.06.066 (2012).
